# Bioactive Phytochemical Constituents of Wild Edible Mushrooms from Southeast Asia

**DOI:** 10.3390/molecules25081972

**Published:** 2020-04-23

**Authors:** Zaw Min Thu, Ko Ko Myo, Hnin Thanda Aung, Marco Clericuzio, Chabaco Armijos, Giovanni Vidari

**Affiliations:** 1 Center of Ningxia Organic Synthesis and Engineering Technology, Institute of Agricultural Resources and Environment, Ningxia Academy of Agriculture and Forestry Sciences, Yinchuan 750002, Ningxia, China; kokomyokalay@gmail.com; 2 Department of Chemistry, Kalay University, Kalay 03044, Sagaing Region, Myanmar; 3 Department of Chemistry, University of Mandalay, Mandalay 100103, Myanmar; hninthandaaung07@gmail.com; 4 DISIT, Università del Piemonte Orientale, Via T. Michel 11, 15121 Alessandria, Italy; marco.clericuzio@uniupo.it; 5 Departamento de Química y Ciencias Exactas, Universidad Técnica Particular de Loja, San Cayetano Alto s/n, Loja 1101608, Ecuador; 6 Medical Analysis Department, Faculty of Science, Tishk International University, Erbil 44001, Kurdistan Region, Iraq

**Keywords:** wild edible mushrooms, Southeast Asia, phytochemical constituents, antioxidant and antimicrobial properties, cytotoxic and immunomodulatory effects

## Abstract

Mushrooms have a long history of uses for their medicinal and nutritional properties. They have been consumed by people for thousands of years. Edible mushrooms are collected in the wild or cultivated worldwide. Recently, mushroom extracts and their secondary metabolites have acquired considerable attention due to their biological effects, which include antioxidant, antimicrobial, anti-cancer, anti-inflammatory, anti-obesity, and immunomodulatory activities. Thus, in addition to phytochemists, nutritionists and consumers are now deeply interested in the phytochemical constituents of mushrooms, which provide beneficial effects to humans in terms of health promotion and reduction of disease-related risks. In recent years, scientific reports on the nutritional, phytochemical and pharmacological properties of mushroom have been overwhelming. However, the bioactive compounds and biological properties of wild edible mushrooms growing in Southeast Asian countries have been rarely described. In this review, the bioactive compounds isolated from 25 selected wild edible mushrooms growing in Southeast Asia have been reviewed, together with their biological activities. Phytoconstituents with antioxidant and antimicrobial activities have been highlighted. Several evidences indicate that mushrooms are good sources for natural antioxidants and antimicrobial agents

## 1. Introduction

Popularly, the term mushrooms (or higher fungi) is used to identify fungi producing macroscopic fruiting bodies. This rather inaccurate definition mostly refers to species belonging to the *phyla* Basidiomycota and Ascomycota. The total number of species of the kingdom fungi is far from being exactly known. It was believed to be around 1.5 millions [1], but more recent estimates have increased the number to a range of 2.2–3.8 millions, worldwide [2]. With 120,000 currently accepted species, it appears that at best just 8% and, in the worst case scenario just 3%, are named so far [2]. The species of fungi described so far are about 120,000. About 35,000 fungal species belong to the *phylum* Basidiomycota, which comprises the majority of “mushrooms” [3].

Mushrooms have been exploited by humans since prehistoric times, both for food and medicinal purposes. About 1069 mushroom species have been reported to be eaten [4]. Indeed, collection and consumption of wild growing mushrooms as a food is a traditional practice in many human cultures. However, cultivated mushrooms are also marketed, and cultivation of mushrooms is increasing everywhere in the world [5]. However, it has been reported that wild mushrooms contain a higher fiber content and more bioactive compounds than cultivated mushrooms [6].

The importance of mushrooms as a food is due not only to their pleasant organoleptic properties but also to the rich content of substances which must be present in a healthy human diet. In fact, mushrooms contain amino acids, fatty acids (many of them being unsaturated or poly-unsaturated, such as oleic, linoleic and linolenic acids), vitamins, sterols, and some essential minerals [7,8,9,10,11,12,13]. As concerns carbohydrates, the most abundant sugar present is trehalose, the α1 → α1 dimer of d-glucose, which is responsible of several alimentary intolerances in people who digest it with difficulty. More elaborate polysaccharides comprise chitin, the fungal *fiber*, which is a homopolymer of *N*-acetylglucosamine and other sugars occurring in fungal cell walls. Notwithstanding the edible properties, a word of warning must be added, about the possibility that toxic metals such as arsenic, mercury, etc. occur in edible mushrooms collected from polluted soils [14,15,16].

In addition as a food, there is an increasing interest in developing mushroom bioactive constituents as control agents of several diseases and to delay aging processes [17,18,19,20]. Friedman, et al. reviewed mushroom polysaccharides which have shown therapeutic properties such as anti-obesity, anti-diabetes, anticancer and antibiotic properties [21]. Mushrooms endowed with potent antimicrobial and antioxidant properties, among other important bioactivities, have been reported in several studies [6,18,22,23,24,25,26].

Little information exists about the phytochemical constituents of edible mushrooms growing in Southeast Asian countries. This review describes the data reported in Reaxys database until January 2020 for some selected edible mushroom growing in Southeast Asia. One purpose of this work is to foster systematic studies on the region’s rich mycological flora.

## 2. Wild Edible Mushroom Species in Southeast Asia 

**Figure 1:**
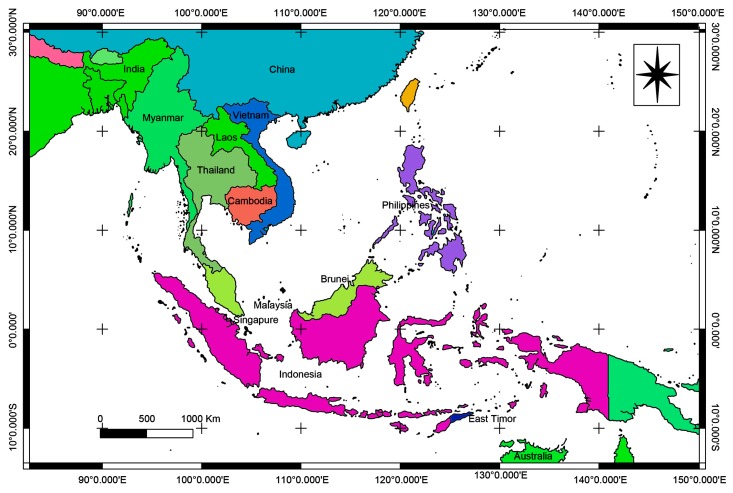
Map showing Southeast Asian countries.

Southeast Asia refers geographically to the corner of Asia east of India, south of China, west of New Guinea, and north of Australia (Figure 1). It is a region with an outstanding high biodiversity, encompassing about 20 percent of global plant, animal and marine species [27,28]. Comparing with the rest of the world, Southeast Asia is more rural as 41.8% of the nearly 590 million people live in the countryside in 2010 [27]. The climate, sea level fluctuations and biotas of this region seem to have created a habit favoring the explosive growth of countless new animal and plants species. In the region surrounding the Mekong river, 2077 new animal and plants species have been described since 1997 and 367 new species were added to the new species record in only two years, 2012–2013 [29]. These findings clearly indicate that the Mekong region has a higher rate of species discovery than other parts of the world.

**Figure 2:**
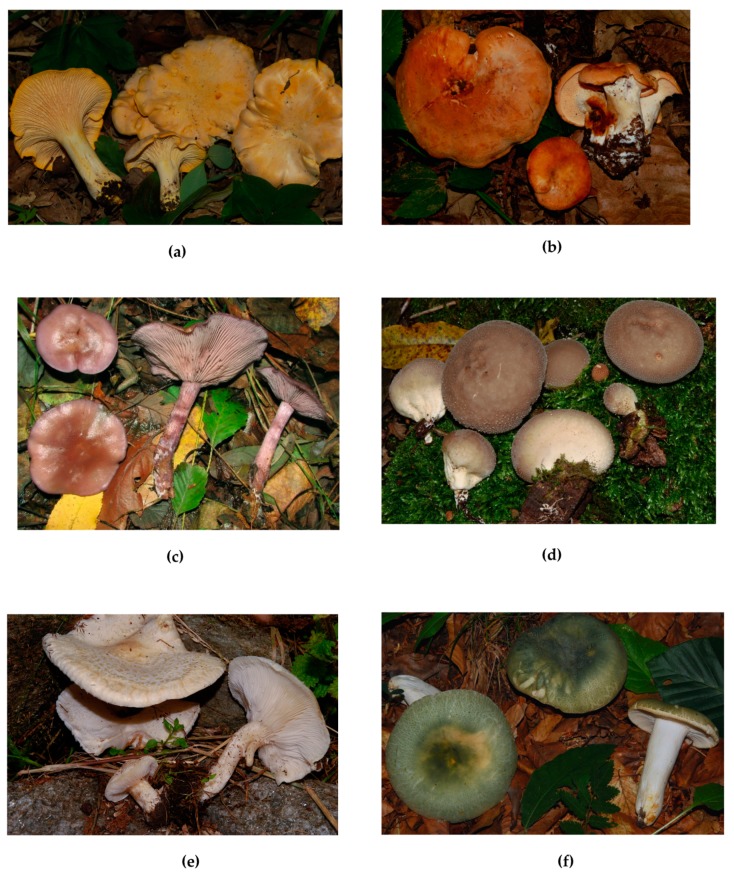
(**a**) *Cantharellus cibarius*; (**b**) *Hydnum repandum*; (**c**) *Lepista sordida*; (**d**) *Lycoperdon pyriforme*; (**e**) *Neolentinus lepideus*; (**f**) *Russula virescens*.

About fungal species, 93% of the fungi growing in northern Thailand appeared to be novel [30]. Therefore, the biodiversity of Southeast Asia is likely to be a vast reservoir for finding new mushroom species, since fungi occurring in Myanmar, Laos, Vietnam, and Cambodia have barely been studied so far [30]. Moreover, it is worth noting that numerous ethnic groups living in Southeast Asian countries resort to several wild mushrooms for obtaining food and medicines; however, very few studies have been carried out on the nutritional value and biological activities of these mushrooms. This review has collected the information available in the literature on the phytochemical constituents of selected wild edible mushrooms occurring in Southeast Asian countries, namely *Agaricus silvaticus*, *Ampulloclitocybe clavipes*, *Butyriboletus roseoflavus*, *Cantharellus cibarius* (Figure 2a), *Craterellus cornucopioides*, *Craterellus odoratus*, *Fistulina hepatica*, *Hydnum repandum* (Figure 2b), *Laccaria amethystea*, *Lactarius hatsudake*, *Lepista sordida* (Figure 2c), *Lycoperdon pyriforme* (Figure 2d), *Neolentinus lepideus* (Figure 2e), *Phlebopus portentosus*, *Polyozellus multiplex*, *Ramaria botrytis*, *Rugiboletus extremiorientalis*, *Russula virescens* (Figure 2f), *Sarcodon imbricatus*, *Termitomyces albuminosus*, *Termitomyces eurhizus*, *Termitomyces heimii*, *Termitomyces microcarpus*, *Thelephora ganbajun*, and *Volvariella bombycina*. These species were selected on the basis of their wide use as a food in Southeast Asia and difficult cultivation. Moreover, they are among the most common mushrooms growing in this part of the world. Likewise all selected macrofungi are a natural resource of economic, ecological, scientific and cultural importance among ethnic groups in Southeast Asia.

**Figure 3:**
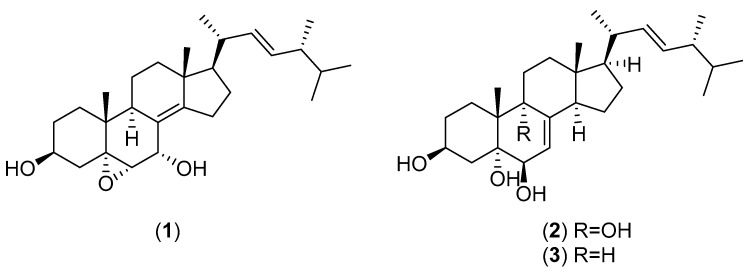
Chemical structures of selected compounds isolated from *Agaricus silvaticus*.

**Figure 4:**
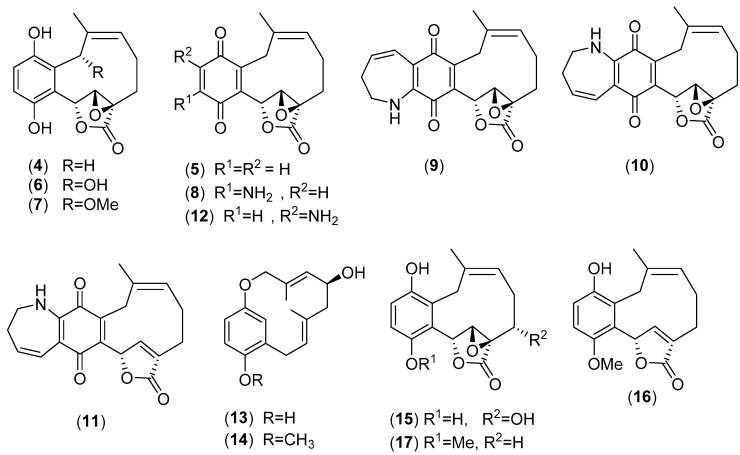
Chemical structures of selected compounds isolated from *Ampulloclitocybe clavipes*.

**Figure 5:**
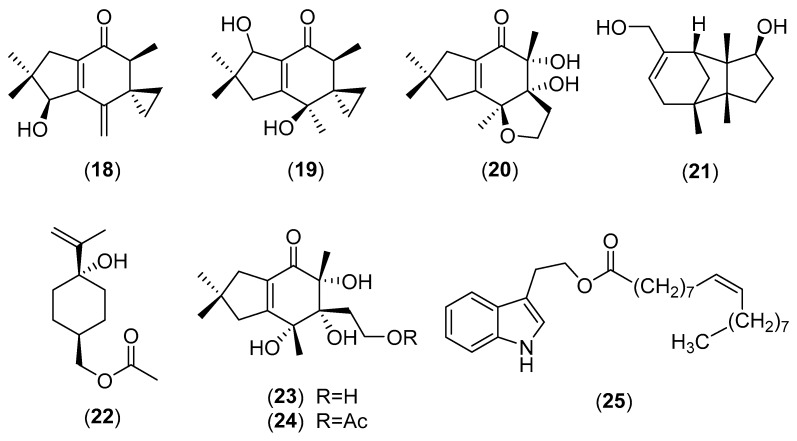
Chemical structures of selected compounds isolated from *Craterellus cornucopioides*.

**Figure 6:**
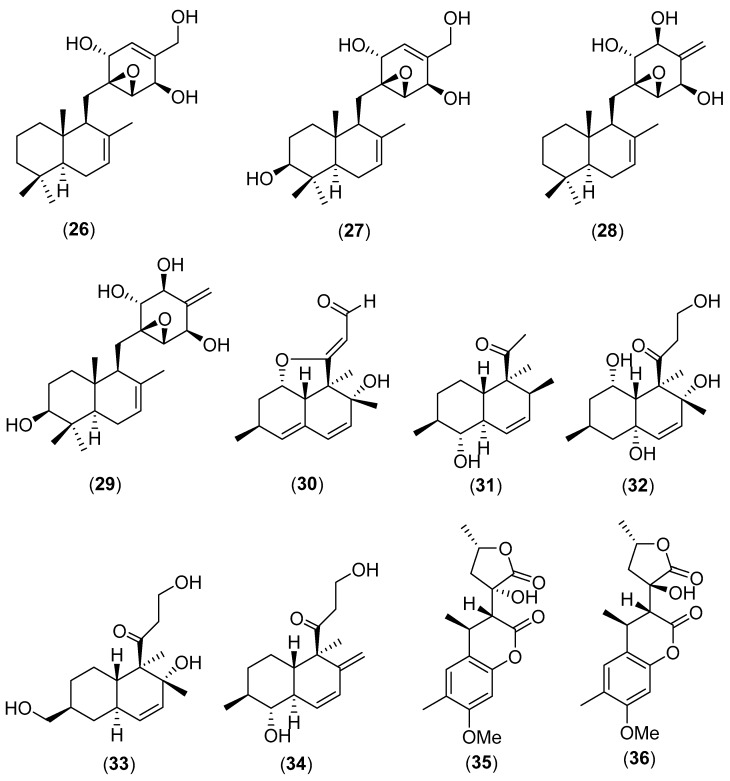
Chemical structures of selected compounds isolated from *Craterellus odoratus*.

**Figure 7:**
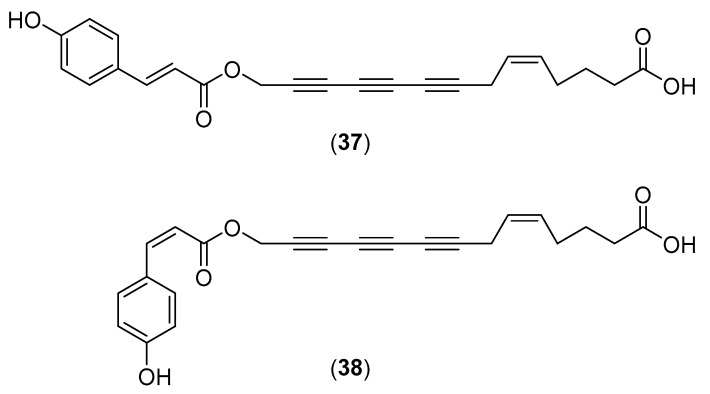
Chemical structures of selected compounds isolated from *Fistulina hepatica*.

**Figure 8:**
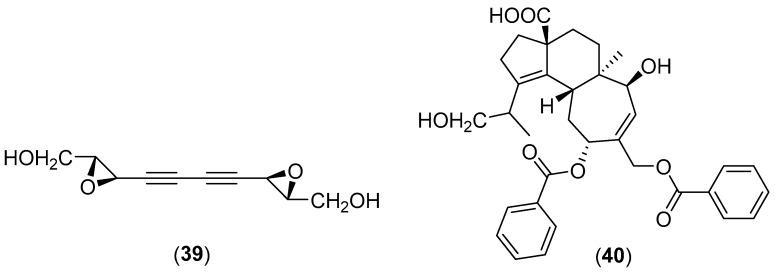
Chemical structures of selected compounds isolated from *Hydnum repandum*.

**Figure 9:**
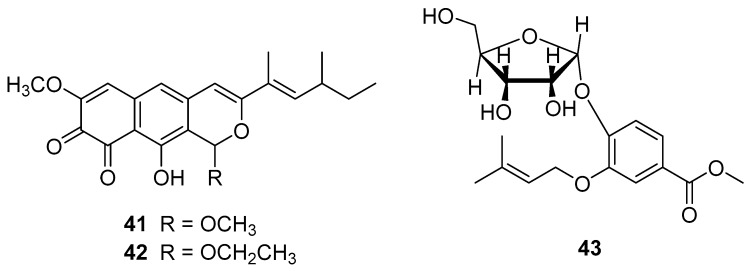
Chemical structures of selected compounds isolated from *Laccaria amethystea*.

**Figure 10:**
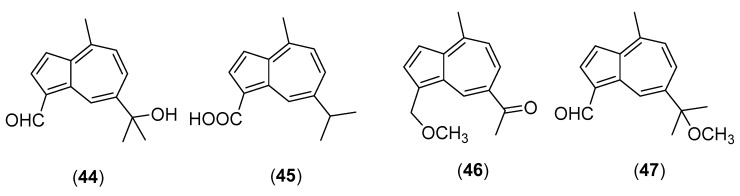
Chemical structures of selected compounds isolated from *Lactarius hatsudake*.

**Figure 11:**
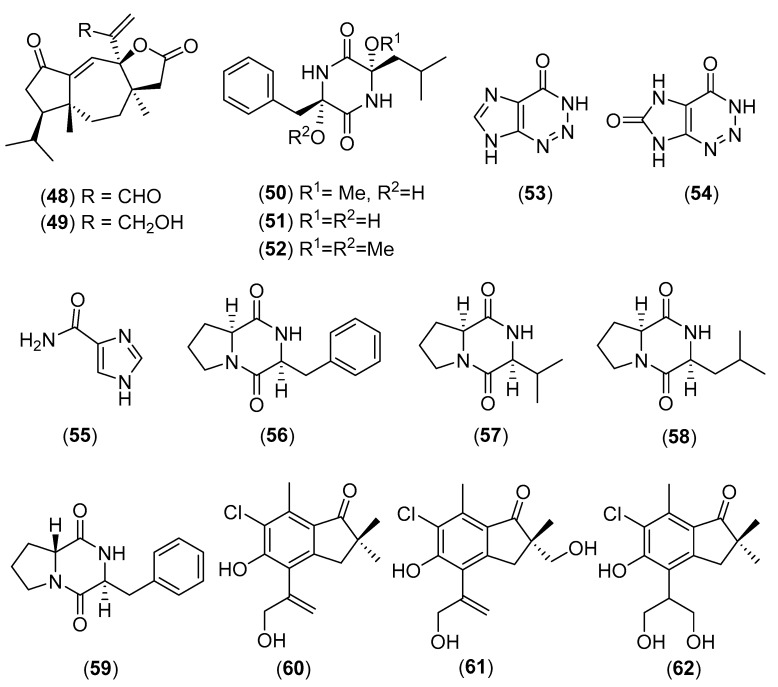
Chemical structures of selected compounds isolated from *Lepista sordida*.

**Figure 12:**
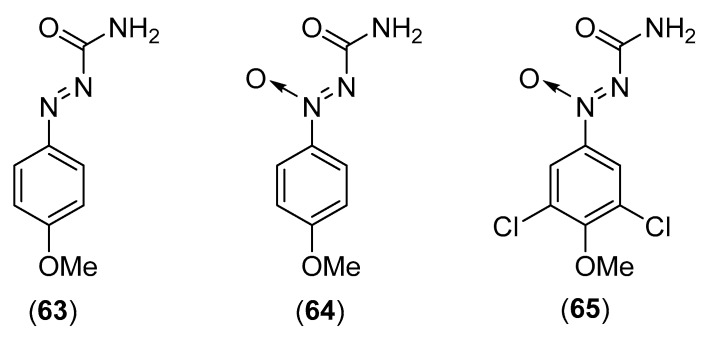
Chemical structures of selected compounds isolated from *Lycoperdon pyriforme*.

**Figure 13:**
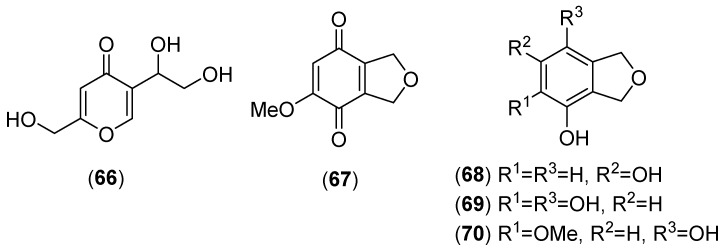
Chemical structures of selected compounds isolated from *Neolentinus lepideus*.

**Figure 14:**
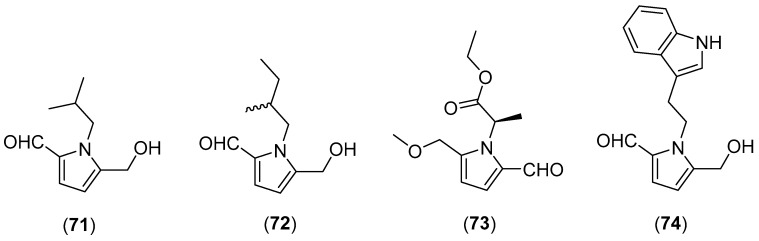
Chemical structures of selected compounds isolated from *Phlebopus portentosus*.

**Figure 15:**
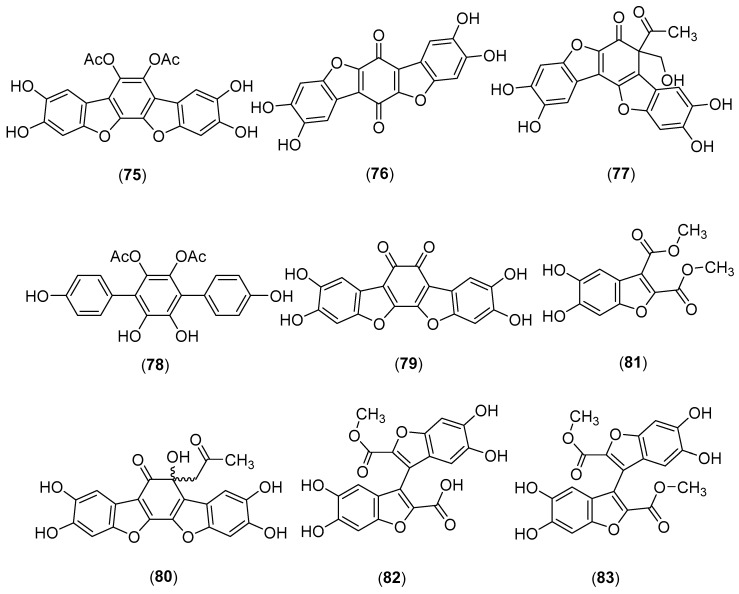
Chemical structures of selected compounds isolated from *Polyozellus multiplex*.

**Figure 16:**
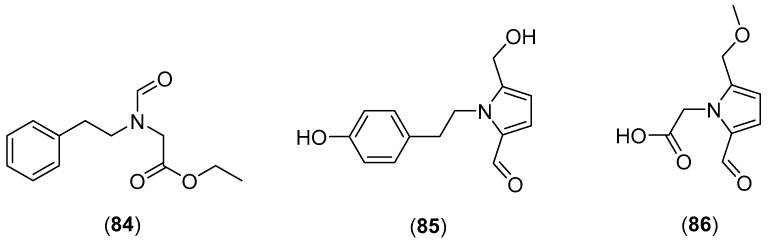
Chemical structures of selected compounds isolated from *Rugiboletus extremiorientalis*.

**Figure 17:**
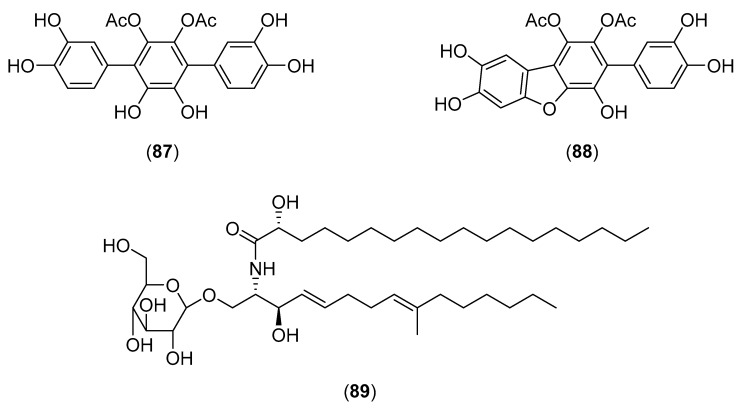
Chemical structures of selected compounds isolated from *Sarcodon imbricatus*.

**Figure 18:**
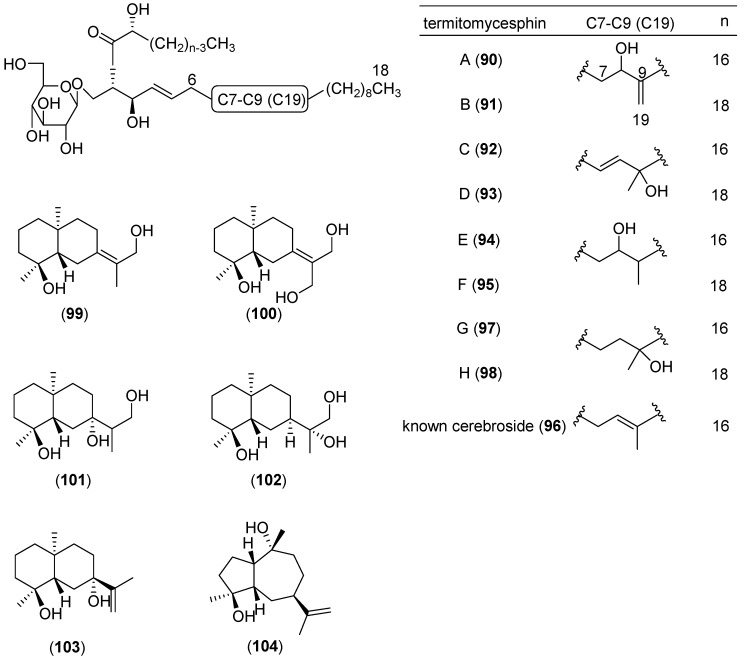
Chemical structures of selected compounds isolated from *Termitomyces albuminosus*.

**Figure 19:**
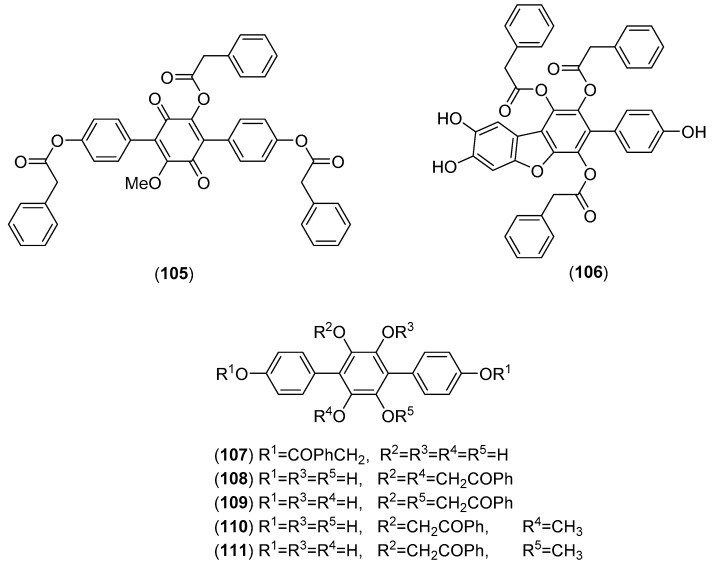
Chemical structures of selected compounds isolated from *Thelephora ganbajun*.

**Figure 20:**
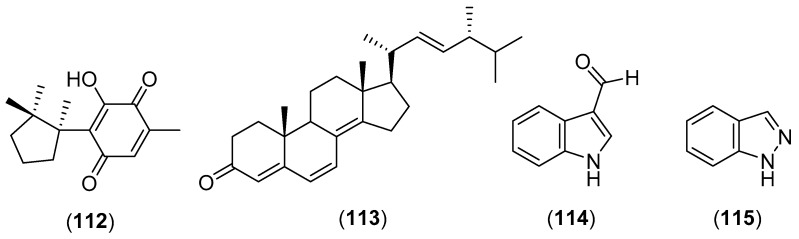
Chemical structures of selected compounds isolated from *Volvariella bombycina*.

The most important biological/pharmacological activities reported for extracts and isolated compounds are also described, with special attention to antioxidant, antimicrobial and cytotoxic properties. They have been summarized in Table 1. The chemical structures of new compounds or compounds that are specific to the collected musroom species are depicted in Figures 3–20. It is worth noting that a great number of data reported herein have been collected through investigations conducted on mushroom samples collected outside Asia, especially in Europe. Therefore, even if the same species is reported to grow in different continents, varieties or sub-varieties may exist for the same species. Thus, possible differences may exist for the phytochemical contents of mushrooms growing in different ecosystems.

## 3. Antioxidant Activity 

Reactive oxygen (ROS) and nitrogen (RNS) species, which are extremely reactive with most organic compounds, are products of the normal cellular metabolism [31] and may have either harmful or beneficial effects on living systems [32]. Free radicals are atoms or molecular fragments containing one or more unpaired electrons in atomic or molecular orbitals [33]. They are formed naturally in the body, especially in mitochondria, as necessary intermediates in a variety of normal biochemical reactions, thus playing a positive role in many normal cellular processes. However, at high concentrations, ROS and RNS are responsible for the oxidative damage to biological macromolecules, including DNA, proteins, and lipids in cell membranes. The damage to cells caused by free radicals, especially the damage to DNA, may contribute to the development of many diseases, including cancer [34,35].

Free-radical scavengers or free-radical quenchers are chemicals that react with free radicals and neutralize them, thus helping stop or limit damages caused by those reactive species. Most cells in our body produce antioxidant and repair systems which protect them against oxidative damage; however, these systems are often insufficient to prevent or repair the damage entirely [36]. Therefore, the introduction in the body of additional antioxidant agents from the diet is believed to be critical for maintaining cell homeostasis and thus a healthy organism [37]. Although synthetic antioxidants such as butylhydroxyanisole (BHA), butylhydroxytoluene (BHT), propyl gallate (PG) and *tert*-butylhydroquinone (TBHQ) have commonly been used as antioxidant additives in foods for years, their safety has long been questioned [38]. This finding has led to an increased interest in natural antioxidants. Antioxidant activities of extracts and isolated compounds from edible mushrooms have been determined by several research groups that used different tests in vitro to measure the reducing power ability, the total antioxidant activity, the 1,1-diphenyl-2-picrylhydrazyl radical scavenging activity, the lipid peroxide inhibitory activity, the ferric reducing antioxidant power, the nitric oxide (NO) scavenging activity, and the ABTS radical scavenging, superoxide radical, and hydroxyl radical scavenging properties. Thus, a large number of results in the literature clearly indicates that several edible mushrooms have significant antioxidant properties due to their bioactive compounds, such as polyphenols, polysaccharides, vitamins, carotenoids and minerals [18,39,40,41].

## 4. Antimicrobial Activity 

Infectious diseases produced by organisms such as bacteria, viruses, fungi or parasites, are among the most serious causes of morbidity and mortality worldwide [42]. Nowadays many infections are often caused by multi-resistant microorganisms resulting in difficult to treat diseases; as a very well-known example, coronavirus Covid-19 is killing thousands of people worldwide. Consequently, healthcare costs are increasing substantially every year, becoming a serious problem in many countries [43,44,45]. This situation has led to an increasing search for new antimicrobial agents from different sources. Several researches have been conducted to explore the antimicrobial potential of natural or synthetic compounds [46,47]. Thus, natural sources, including mushrooms, have been investigated for finding novel antimicrobial compounds [48,49,50,51].

In food industry, contamination of food products by bacteria and fungi may be the result of exposure to sources of contamination during harvesting, processing and/or packaging process [52]. Therefore, chemical additives have been extensively used in food industries to increase the shelf life of food and to prevent the proliferation of microorganisms. In this regard, natural antimicrobials, including those isolated from mushrooms, are gaining an increasing importance as potential alternatives to synthetic preservatives, whose safety and impact on human health are still questionable [53,54,55]. Instead, the safety of many natural antimicrobials have been generally recognized in EU and USA [56].

## 5. Bioactive Phytochemical Constituents of Wild Edible Mushrooms from Southeast Asian Countries

### 5.1. Agaricus silvaticus Schaeff.

*Agaricus silvaticus* Schaeff. is a common edible mushroom belonging to the family Agaricaceae. It is distributed in China, Thailand and Mongolia [4,57,58]. Boonyanuphap and Hansawasdi studied the beta-glucan content of *A. silvaticus* comparing it with other wild edible mushrooms found in Thailand [59]. β-Nitroaminoalanine, *N*-nitroethylenediamine and glutamic acid were identified as secondary metabolites of *A. silvaticus* [60,61,62]. Lodonjav et al. [57] investigated the chemical components of *A. silvaticus* and identified 5α,6α-epoxy-(22*E*,24*R*)-ergosta-8(14),22-diene-3β,7α-diol (**1** in Figure 3), ergosterol, ergosterol peroxide, (22*E*,24*R*)-ergosta-7,22-diene-3β,5α,6β,9α-tetraol (**2**), cerevisterol (**3**), (2*R*,3*S*,4*R*,6*E*)-*N*-[(*R*)-2′-hydroxytetracosanoyl]-1,3,4-trihydroxy-2-amino-octadeca-6-ene, benzoic acid, cinnamic acid and d-mannitol. The antimicrobial activity of *A. silvaticus* has not been reported, whereas the antioxidant activity has been determined [63,64].

### 5.2. Ampulloclitocybe clavipes (Pers.) Redhead, Lutzoni, Moncalvo and Vilgalys

*Ampulloclitocybe clavipes* (Pers.) Redhead, Lutzoni, Moncalvo & Vilgalys, formerly known as *Clitocybe clavipes* (Pers.) P. Kumm, belongs to the family Hygrophoraceae. It is a wild mushroom growing in China, Thailand, and Japan [4,58,65]. Clavilactones A–C (**4**–**6**, Figure 4) were isolated in an Italian laboratory from a culture of the fungus and exhibited antifungal and antibacterial activities [66]. The structures of clavilactones D and E (**7**), were initially inferred by 1- and 2-D NMR data [67]. However, the subsequent total synthesis of clavilactones A, B, and D led to a revision of the original structure of clavilactone D which was established to be as formula (**8**) in Figure 4 [68]. Clavilactone A, B, and D displayed potent inhibitory activity in kinase assays against the Ret/ptc1 and epidermal growth factor receptor (EGFR) tyrosine kinases [67,69]. Subsequently, Sun et al. [70] isolated from a fungal strain of *A. clavipes*, clavilactone F (**12**) together with three novel meroterpenoids, named clavipines A–C (**9**–**11**), which exhibit a benzoquinone ring fused to an azepine ring and a ten-membered carbocycle bearing an α,β-epoxy/unsaturated-γ-lactone. Compound **9** exhibited significant antiproliferative activity against HepG2 and A549 cells with IC_50_ values of 4.28 ± 0.26 and 7.49 ± 0.41 μM, respectively [70]. Subsequently, clavipols A–B (**13**–**14**) containing a 12-membered ether ring and clavilactones G–I (**15**–**17**) were isolated from the fruiting bodies of *A. clavipes* collected in China. Compound **16** exhibited moderate cytotoxic activity against Hela and SGC-7901 cancer cell lines, with IC_50_ values of 23.5 and 14.5 µM, respectively [71]. Five fatty acid derivatives, isolated from *A. clavipes* have been reported to have potent strong inhibitory activity against aldehyde dehydrogenase [65].

### 5.3. Butyriboletus roseoflavus (M. Zang and H.B. Li) D. Arora and J.L. Frank

*Butyriboletus roseoflavus* is an Asian species which was previously named *Boletus speciosus* Frost. It belongs to the family Boletaceae and to the genus *Butyriboletus*, which has recently been created to accommodate the former section *Appendiculati* within the large genus *Boletus*. This edible mushroom grows abundantly in Southern China (Yunnan) and Thailand, and it is commonly sold in street markets [4,58]. A group of Chinese researchers reported the isolation, from the fruiting bodies of a novel heteropolysaccharide, which has a backbone of (1→4)-α-l-mannopyranose residues, which branched at *O*-6. The branches are mainly composed of one with →1)-α-d-galactopyranose residue [72]. In addition to a strong antioxidant activity [72], this polysaccharide with a unique structure activates the secretion of cytokines from immune cells and inhibits the growth of Hep-2 cells. The concentration of 400 μg/mL has the highest inhibitory rate [73,74]. A new water-soluble polysaccharide, having a backbone of 1,4-linked β-d-glucose, with branches mainly composed of two 1,6-linked α-d-galactose residues and bearing a 4-linked β-d-glucose unit at the end of the branches, has been reported to exhibit unique antitumor and immunoregulatory properties [75]. Sun et al. [76] reported that hemagglutinin isolated from *B. speciosus*, showed antiproliferative activity towards hepatoma Hep G2 cells and mouse lymphocytic leukemia cells (L1210) in vitro, with an IC_50_ of 4.7 μM and 7.0 μM, respectively. It also exhibited HIV-1 reverse transcriptase inhibitory activity with an IC_50_ of 7.1 μM.

### 5.4. Cantharellus cibarius Fr.

*Cantharellus cibarius* Fr., belonging to the family Cantharellaceae, is an edible mushroom, which grows widely in China, India, Thailand, America and several European countries [4,58,77,78,79]. The polysaccharides isolated from the fruiting bodies of *C. cibarius* were galactans and glucans, including a novel linear 3-*O*-methylated galactan and a new heteropolysaccharide. These macromolecules showed a wide range of biological activities, such as antioxidant, antitumor, antiproliferative, immunomodulatory and neuroprotective properties [80,81,82,83,84,85,86]. Mittermeier et al. [87] investigated the taste active and taste modulating compounds from this mushroom by LC–MS and 1D/2D-NMR experiments and identified several C18-acetylenic acids: 14,15-dehydrocrepenynic acid methyl ester, 14,15-dehydrocrepenynic acid ethyl ester, 14,15-dehydrocrepenynic acid, (9*Z*,15*E*)-14,17,18-trihydroxy-9,15-octadecadien-12-ynoic acid, (9*Z*,15*E*)-14-oxo-9,15-octadecadien-12-ynoic acid, (10*E*,15*E*)-9-hydroxy-14-oxo-10,15-octadecadien-12-ynoic acid, (10*E*,15*E*)-9-hydroperoxy-14-oxo-10,15-octadecadien-12-ynoic acid, (10*E*,15*E*)-9,14-dioxo-10,15-octadecadien-12-ynoic acid, (9*Z*,15*E*)- 14-oxo-9,15-octadecadien-12-ynoic acid methyl ester, (9*Z*,15*E*)-17(18)-epoxy-14-oxo-9,15-octadecadien-12-ynoic acid methyl ester, (10*E*,14*Z*)-9-hydroperoxy-10,14-octadecadien-12-ynoic acid, (10*E*,14*Z*)-12-hydroxy-10,14-octadecadienoic acid, (9*Z*,11*Z*)-14,18-dihydroxy-9,11-octadecadienoic acid, (9*Z*,11*Z*)-14,17,18-trihydroxy-9,11-octadecadienoic acid, (10*E*,14*Z*)-9-hydroxy-10,14-octadecadien-12-ynoic acid and (10*E*,14*Z*)-9-oxo-10,14-octadecadien-12-ynoic acid. Further studies showed that *C. cibarius* also contains (9*Z*,13*Z*,15*E*)-14,18-dihydroxy-12-keto-9,13,15-octadecatrienoic acid, 14,15-dehydrocrepenyic acid, (10*E*,14*Z*)-9-oxooctadeca-10,14-dien-12-ynoic acid and (10*E*,14*Z*)-9-hydroxyoctadeca-10,14-dien-12-ynoic acid and ergocalciferol [88,89,90]. Crude extracts of *C. cibarius* showed antioxidant [91], antimicrobial activity [92] and cytotoxic activities [93].

### 5.5. Craterellus cornucopioides (L.Fr.) Pers

*Craterellus cornucopioides* (L.Fr.) Pers. (family-Cantharellaceae) is an edible fungus with a wide distribution in Europe, North America, Korea, Japan, China, and Thailand [4,58,94,95]. A new triple-helix polysaccharide, a heteroglycan with (1→3)-linked-β-d-Man*p*-(1→6)-linked α-d-Gal*p* backbone distributed by (1→4)-linked-α-d-Xyl*p*-t-α-d-Man*p* and t-β-d-Glu*p* units at *O*-6, was isolated from *C. cornucopioides*. This compound activated RAW264.7 macrophages in vitro, and enhanced the immunomodulatory activity in immunosuppressive mice models [94,95,96]. Yang et al. [97] isolated a novel polysaccharide fraction from the fruiting bodies. The dominant linkage types were →3,6)-Man*p* (1→, T-Ara*f*, →4,6)-Man*p* (1→, →5)-Ara*f* (1→ and →3)-Ara*f* (1→). The polysaccharide possessed strong scavenging abilities on DPPH and ABTS radicals. Three illudin sesquiterpenoids, craterellins A–C (structures **18**–**20** in Figure 5), and one gymnomitrane sesquiterpenoid, gymnomitr-3-en-10β,15-diol (**21**), together with illudin F, illudin M, illudin T and illudalenol were isolated in China from cultures of this mushroom. Compound **20** exhibited moderate cytotoxicity against A-549 cells with an IC_50_ value of 21.0 μM [98]. In addition to a new menthane monoterpene, 4-hydroxy-4-isopropenylcyclohexanemethanol acetate (**22**), craterellins D (**23**) and E (**24**) were later isolated from fungal cultures after minor modifications of the original cultural conditions. The cytotoxic activities of these compounds on five tumor cell lines were also reported [99]. Three new keto esters, 4-oxo-hex-1,6-diyl diacetate, 4-oxo-hex-5-enyl acetate and 6-hydroxy-4-oxo-hexyl acetate were isolated from a tissue culture of fruiting bodies of *C. cornucopioides* collected in China [100]. Magnus’s group isolated three tryptophol (indole-3-ethanol) derivatives, namely 2-(indol-3-yl)ethyl octadeca-(9*Z*)-enoate (structure **25** in Figure 5), 2-(indol-3-yl)ethyl octadeca-(9*Z*,12*Z*)-dienoate and 2-(indol-3-yl)ethyl octadeca-(9*Z*,14*Z*)-dien-12-ynoate from the fruiting bodies of this mushroom [101]. Glycerol *tri*-dehydrocrepenynate, glycerol trioleate and glycerol linoleate dioleate were also isolated by the same research group [102]. Piceatannol, vitamin B_12_, ergosterol and ergosteryl derivatives are other chemical constituents isolated from *C. cornucopioides* [93,103,104]. Various extracts of *C. cornucopioides* showed antioxidant, antimicrobial, anti-inflammatory and cytotoxic activities [91,93,105,106,107,108].

### 5.6. Craterellus odoratus (Schwein.) Fr.

*Craterellus odoratus* (Schwein.) Fr. is a tasty mushroom of the family Cantharellaceae, which is widely collected in China and Thailand [58,109,110]. Three rare merosesquiterpenoids, named craterellins A–C (**26**–**28** in Figure 6), were isolated from cultures of *C. odoratus* together with known massarinolin C. They showed inhibitory activities of 11β-hydroxysteroid dehydrogenases (11β-HSD1 and 11β-HSD2) [111]. Craterellin A (**26**) demonstrated significant a inhibitory activity against human 11β-HSD2 with an IC_50_ value of 1.5 µg/mL [111]. Craterellin D (**29**), 5-hydroxymethyl-2-hydroxy-4-methoxy-phenylethanone, 2-(1,2-dihydroxypropan-2-yl) benzofuran-5-carboxylic acid, 6α-hydroxy-3-methoxy-4α-methyl-2-cyclohexen-1-one have been isolated from the cultures of *C. odoratus*. 5-hydroxymethyl-2-hydroxy-4-methoxy-phenylethanone exhibited inhibitory activity against human 11β-HSD1 with an IC_50_ value of 16.4 µg/mL [109]. Guo et al. [112] extensively studied the cultures of *C. odoratus* and identified five new polyketides, named craterellones A–E (structures **30**–**34** in Figure 6), together with the known compounds decumbenones A and B, versiol, calbistrin A and calbistrin C. Their cytotoxic activities were reported [112]. Subsequently, the Chinese research group reported the chemical structures of two rare 4,6-dimethyl-3,4-dihydrochromen-2-one derivatives, cralactones A (**35**) and B (**36**), which were isolated from the culture broth of *C. odoratus*. The pancreatic lipase inhibitory activity of the compounds were also described [113]. Recently, the origin of these isolated compounds has been discussed. In fact, it has been debated if they are true metabolites of *C. odoratus* or are formed by the associated fungus *Montagnula donacina* [114].

### 5.7. Fistulina hepatica (Schaeff.)

*Fistulina hepatica* (Schaeff.), commonly known as beefsteak fungus, is a wild edible fungus belonging to the family Fistulinaceae [95]. It is distributed in temperate and subtropical hardwood forests of China, Thailand, Hungary, Portugal [4,58,115,116], and other European countries. Two novel triacetylene derivatives have been isolated from the fruiting bodies and named cinnatriacetins A (**37**) and B (**38**) [117]. Compounds **37** and **38** (see structures in Figure 7) showed antimicrobial activity against gram-positive bacteria, but no activity towards gram-negative bacteria [117]. Caffeic acid, *p*-coumaric acid, ellagic acid, hyperoside, quercetin, oxalic acid, aconitic acid, citric acid, malic acid, ascorbic acid and fumaric acid were also isolated from *F. hepatica*, and an aqueous extract showed a significant scavenger activity of DPPH^•^ and superoxide radicals [118]. A sample of *F. hepatica* collected in Portugal contained tocopherols and showed strong antioxidant activity [115,119]. Ribeiro and his co-workers extensively studied the free amino acid and fatty acid composition of *F. hepatica,* comparing their contents with those of other wild edible mushrooms [120,121]. Wu et al. [122] studied the volatile compounds from the fruiting bodies and 11 compounds were identified as responsible for the characteristic odor of the fungus. They were: 1-octen-3-one, 1-octen-3-ol, linalool, phenylacetaldehyde, butanoic acid, (*E*)-2-methyl-2-butenoic acid, methyl (*E*)-cinnamate, (*Z*)-9-hexadecenoic acid methyl ester, bisabolol oxide B, phenylacetic acid, and an undetermined mouldy compound. (*E*)-2-Methyl-2-butenoic acid and bisabolol oxide B have not been identified as native fungal volatile metabolites. Other studies on the volatiles from *F. hepatica* have been performed in Portugal and German laboratories [123,124]. A methanol/water (80:20) extract of *F. hepatica* collected in Portugal inhibited the growth of gram-negative (*Escherichia coli*, *Morganella morganni* and *Pasteurella multocida*) and gram-positive (*Staphylococcus aureus*, MRSA, *Enterococcus faecalis*, *Listeria monocytogenes*, *Streptococcus agalactiae* and *Streptococcus pyogenes*) bacteria [22]. Moreover, the crude extract showed high synergistic effects in combination with cefuroxime against MRSA [116,125].

### 5.8. Hydnum repandum L.

*Hydnum repandum* L. is a wild edible mushroom belonging to the family Cantharellaceae [95]. This mushroom is distributed in China, Thailand, India and Portugal [58,126,127,128]. A new cytotoxic diepoxide, namely repandiol (structure **39** in Figure 8), was isolated from fruiting bodies collected in Japan and displayed potent cytotoxic activity against various tumor cell lines, especially colon adenocarcinoma cells with an IC_50_ value of 0.30 µg/mL [129]. Sarcodonin A, scabronine B (**40**), 3β-hydroxy-5α,8α-epidioxyergosta-6,22-diene, (22*E*,24*R*)-ergosta-7,22-diene-3β,5α,6β-triol, (22*E*,24*R*)-ergosta-7,22-diene-3β-ol, benzoic acid, 4-hydroxylbenzaldehyde, 4-monopropanoylbenzenediol, ethyl-β-d-glucopyranoside, thioacetic anhydride, and (2*S*,2’*R*,3*S*,4*R*)-2-(2-hydroxytricosanoylamino) hexadecane-1,3,4-triol have also been isolated [130]. Fatty acids such as pentadecanoic, heptadecanoic, oleic, myristoleic, palmitoleic, linolenic, palmitic and stearic acids were detected in the fruiting bodies of *H. repandum* collected in India [128]. Antioxidant, antiproliferative, cytotoxic, and pro-apoptotic activities of *H. repandum* were investigated by Vasdekis and collaborators. A significant cytotoxicity (IC_50_ = 1.0 mg · mL^−1^) was determined against an A549 cell line, and, piceatannol was identified by LC/MS and MS analysis [93]. The influence of *H. repandum* extract on the growth and sporulation of *Penicillium expansum* was studied in vitro. A significant reduction of the mycelial growth and inhibition of the pathogen sporulation were observed [131]. *In vitro* antimicrobial and antioxidant susceptibility studies were performed by many research groups [92,115,126,127,132,133].

### 5.9. Laccaria amethystea (Bull.) Murrill

*Laccaria amethystea* (Bull.) Murrill, belonging to the family Hydnangiaceae, is an edible mushroom with a wide distribution in China, Thailand and Laos [4,58]. Berg et al. [134] reported the isolation from a strain of *L. amethystea*, of new protease inhibitors, called laccaridiones A and B (structures **41** and **42**, respectively, in Figure 9), which inhibited a series of proteases such as commercial trypsin, papain, thermolysin, collagenase, and zinc-protease from *Bacillus subtilis*. In addition, compound **42** showed strong antiproliferative effects on the murine fibroblast-cell line L-929 (IC_50_ = 2.4 µg/mL) and the human leukemia cell line K-562 (IC_50_ = 1.8 µ/mL) [134]. 3-(3-Methylbut-2-enyloxy)-4-*O*-α-d-ribofuranosyl-benzoic acid methyl ester (**43**), was also isolate from a culture of this mushroom [135]. *L. amethystea* showed effective anti-hyperglycemia and anti-oxidative properties; the highest α-amylase inhibitory activity (EC_50_ value 4.37 µg/mL) and metal chelating activity (EC_50_ value 2.13 mg/mL) were observed for an aqueous extract [106].

### 5.10. Lactarius hatsudake Nobuj. Tanaka

*Lactarius hatsudake* Nobuj. Tanaka, belonging to the genus *Lactarius* of the family Russulaceae, is an edible, slightly bitter mushroom, which is widely distributed in China, Thailand and Bhutan [4,58]. Artificial cultures are obtained with difficulty [136]. A review on the secondary metabolites isolated from the fruiting bodies of European *Lactarius* species [137] does not include this mushrooom, which is a typical Asian species for which a limited number of reports exists. Miyazawa et al. [138] studied the components of the volatile oil from this mushroom. *cis*-Isolongifolanone, α-cedrene epoxide, humulene epoxide III, clovane, linoleic acid and palmitoleic acid were the main components among the 71 identified compounds. Ergosterol, ergosterol peroxide, 5α,8α-epidioxy-(24*S*)-ergosta-6-en-3β-ol and (22*E*,24*R*)-ergosta-7,22-dien-3β,5α,6β-triol were isolated from the fruiting bodies, and their inhibitory activitities against *Crotalus adamenteus* venom phospholipase A_2_ (PLA_2_) enzyme and HIV in vitro were reported [139,140]. Fang et al. [141] isolated 7-(1-hydroxy-1-methylethyl)-4-methylazulene-1-carbaldehyde (structure **44** in Figure 10), 4-methyl-7-(1-methylethyl) azulene-1-carboxylic acid (**45**) and 4-methyl-7-(1-methylethyl)azulene-1-carbaldehyde from the fruiting bodies. Other new guaiane sesquiterpenes, called lactariolines A and B (structures **46** and **47**, respectively, in Figure 10), together with known 4-methyl-7-isopropylazulene- 1-carboxylic acid, 1-formyl-4-methyl-7-isopropyl azulene, lactaroviolin and 1-formyl-4-methyl-7-(1-hydroxy-1-methylethyl) azulene, were isolated by a Korean research group [142].

### 5.11. Lepista sordida (Schumach.) Singer

*Lepista sordida* (Schumach.) Singer, a basidiomycetous fungus of the family Tricholomataceae, is an edible and medicinal agaric species which grows in the wild in China, Thailand, Korea [4,58,143]. Moreover, there is a report on the artificial cultivation of a wild strain of *L. sordida* from Thailand [144]. A water-soluble polysaccharide isolated from the fruiting bodies, which significantly increased the nitric oxide and NF-α release from macrophages, was established to have a backbone consisting of (1→6)-linked-α-d-glucopyranosyl and (1→2,6)-linked-α-d-glucopyranosyl residues, terminated with a terminal (1→)-α-d-galactopyranosyl residue at the *O*-3 position of a (1→2,6)-linked-α-d-glucopyranosyl residue along the main chain [145]. Miao and co-worker extracted four water-soluble polysaccharides from the fruiting bodies which showed potent antiproliferative effects on human laryngocarcinoma Hep-2 cells in vitro and in vivo [146,147]. Intracellular polysaccharides from mycelium of *L. sordida* have demonstrated to possess a significant free radical-scavenging activity in vitro on hydroxyl, superoxide anion and DPPH radicals [148]. Two new diterpenoids, lepistal and lepistol (structures **48** and **49**, respectively in Figure 11), were isolated from fungal fermentations of *L. sordida* collected in France [149]. Aldehyde **48** was more active than alcohol **49** as regards the cytotoxic, antibacterial and antifungal activities [149]. Compounds **50**–**52** (see structures in Figure 11**)**, named lepistamides A–C, were also isolated, in conjunction with diatretol, from samples of *L. sordida* collected in China [150]. A group of Japanese researchers isolated plant-growth regulating compounds, 2-azahypoxanthine (**53**), 2-aza-8-oxohypoxanthine (**54**), and imidazole-4-carboxamide (**55**) [151,152,153,154], whereas compounds **56**–**59** (see structures in Figure 11**)**, showing inhibitory activity of the bentgrass root growth, were isolated from a culture broth [155]. The isolation of three new chlorinated sesquiterpenes from a culture broth of *L. sordida*, named lepistatins A–C (see structures **60**–**62** in Figure 11), was reported by a Korean research group along with their antibacterial and antiproliferative activities [143]. In conclusion, polysaccharides from *L. sordida* were determined to possess immunoregulatory [145], antiproliferative [146], anticancer [146,147], and antiradical activities [148], while different secondary metabolites showed antimicrobial [149], cytotoxic [149], and plant growth regulatory activities [151,152,153,154].

### 5.12. Lycoperdon pyriforme Schaeff.

*Lycoperdon pyriforme* Schaeff., belonging to the family Agaricaceae, is a wild edible mushroom which grows in China, Thailand, Turkey and Bulgaria [4,58,156,157]. Akatin reported the isolation and characterization of a new β-glucosidase [157]. Another research group isolated 4-methoxy-benzene-1-azoformamide (**63**), 4-methoxybenzene-1-*ONN*-azoxyformamide (**64**) and 3,5-dichloro-4-methoxybenzene-1-*ONN*-azoxyformamide (**65**) [158]. Compounds **63** and **64** (see structures in Figure 12) were active against the plant parasitic nematode *Meloidogyne incognita*, and showed weak antimicrobial effects against *Nadsonia fulvescens* and *Penicillium notatum*. Compound **65** (see structure in Figure 12) exhibited weak cytotoxicity against L1210, HL-60, and HeLa S3 cells [158]. *L. pyrifonne* has also been reported to contain linoleic, oleic, palmitic, stearic, 9-eicosenoic, 9,12-eicosadienoic, tricosanoic, pentacosanoic, hexacosanoic, and 11-hexacosenoic acids [156]. Biological studies were conducted on the antioxidant and antimicrobial activities of *L. pyrifonne* [159,160,161].

### 5.13. Neolentinus lepideus (Fr.) Redhead and Ginns

*Neolentinus lepideus* (Fr.) Redhead & Ginns, belonging to the family Polyporaceae, was previously named *Lentinus lepideus*. It grows in China, Thailand, Japan and Korea [4,58,162,163]. It is worth noting that while some authors qualify this mushroom as edible, others describe it as inedible. Hanssen extensively studied the liquid cultures and reported the presence of (−)-torreyol, (−)-T-muurolol, (+)-T-cadinol, (−)-α-cadinol, cubenol, epicubenol, *trans*,*trans*-farnesol, drimenol, α-copaene, α-elemene, *trans*-β-farnesene, γ-muurolene, α-muurolene, δ-cadinene, cadina-1,4-diene and calacorene [164,165]. A new γ-pyrone derivative, named lepidepyrone (see structure **66** in Figure 13), together with methyl 3-hydroxy-4-methoxycinnamate and ergosterol were isolated from the cultured mycelium of the mushroom. Compound **66** showed high inhibitory activity on mammalian HAase with an IC_50_ = 3.3 mM [162]. Phytochemical investigations of *N. lepideus* established the presence in the fruiting bodies of two new secondary metabolites, 5-methoxyisobenzofuran-4,7(1*H*,3*H*)-dione (**67**) and 1,3-dihydroisobenzofuran-4,6-diol (**68**), together with the known compounds 5-methoxy-2,3-dimethylcyclohexa-2,5-diene-1,4-dione, (*E*)-3-(3-methoxyphenyl)acrylic acid, 3-(4-methoxyphenyl)propan-1-ol, (*E*)-3-(4-methoxyphenyl)acrylic acid, methyl (*E*)-3-(2-methoxyphenyl)acrylate, methyl (*E*)-3-(3-hydroxy-4-methoxyphenyl)acrylate, and methyl (*E*)-3-(4-methoxyphenyl)acrylate [166]. Compounds **67** and **68** (see structures in Figure 13) showed nitric oxide inhibitory activity with IC_50_ values of 6.2 µM and 88.8 µM, respectively. In addition, compound **68** displayed antioxidant activity with an IC_50_ value of 68.6 µM [166]. 1,3-Dihydroisobenzofuran-4,5,7-triol (**69**) and 5-methoxy-1,3-dihydroisobenzofuran-4,7-diol (**70**) were isolated from an EtOAc extract of a culture filtrate and showed tyrosinase inhibitory activity with IC_50_ values of 173 and 263 μg/mL, respectively [167]. Extracts from the fruiting bodies of *N. lepideus* have been reported to possess antioxidant [168], antityrosinase [168], antihyperlipidemic [163], and immunomodulating activities [169,170].

### 5.14. Phlebopus portentosus (Berk. & Broome) Boedijn

*Phlebopus portentosus* (Berk. & Broome) Boedijn, belonging to the family Boletinellaceae, is a popular edible mushroom in China and Thailand [171]. Although this mushroom grows wild in association with hosts in mixed forests and orchards, nowadays it can be grown in artificial cultures [171,172]. Kaewnarin et al. [173] evaluated the antioxidant, anti-tyrosinase, and antihyperglycaemic activities of *P. portentosus* as well as the phenolic content, comparing it with other three wild edible mushrooms. Three novel pyrrole alkaloids, named phlebopines A–C (structures **71**–**73** in Figure 14), together with four known ones, 2-[2-formyl-5-(methoxymethyl)-1*H*-pyrrole-1-yl]propanoate, inotopyrrole, 1-isopentyl-2-formyl-5-hydroxy-methylpyrrole and inotopyrrole B (**74**), were isolated from fruiting bodies collected in China. Among these isolated compounds, inotopyrrole B (**74**) displayed remarkable neuroprotective effects against hydrogen peroxide-induced neuronal-cell damage in human neuroblastoma SH-SY5Y cells [174].

### 5.15. Polyozellus multiplex (Underw.) Murrill

*Polyozellus multiplex* (Underw.) Murrill, belonging to the family Thelephoraceae, grows in the wild in Japan, Korea, China, and Thailand [4,58,175]. A new inhibitor of prolyl endopeptidase (PEP) with an IC_50_ value of 2.72 µM, named polyozellin, was identified from a methanolic extract of fresh fruiting bodies collected in Korea [176]. The total synthesis of polyozellin by Takahashi and his collaborators led to a revision of the structure which was determined to be **75** [177]. A Korean research group investigated the EtOAc soluble fraction of the mushroom and reported the chemical structure of two active compounds, thelephoric acid (**76**) and kynapcin-9 (**77**) with their PEP activities [178]. Another *p*-terphenyl derivative, named kynapcin-12, having PEP inhibitory activity with an IC_50_ value of 1.25 µM, was isolated by Lee and collaborators from a methanolic extract [179]. The correct chemical structure of kynapcin-12 (**78**) was later assigned by total synthesis [180]. Polyozellic acid (**79**), and the acetone adduct (**80**), together with thelephoric acid, were isolated from *P. multiplex* collected in Japan and showed inhibitory effects on the proliferation, tubule formation, and invasion of human umbilical vein endothelial cells [181]. Compounds **75**, **76**, **78**, and **79** (see structures in Figure 15) inhibited BACE1 activity with IC_50_ values of 3.08, 3.50, 4.78, and 15.79 μM, respectively, and neuroprotective activities in glutamate-induced HT22 cell death [175]. Kim et al. [182] reported the isolation of two new benzofurans, named kynapcin-13 (**81**) and kynapcin-28 (**82**), from *P multiplex*, which inhibited prolyl endopeptidase with IC_50_ values of 76.80 and 0.98 μM, respectively. Another new benzofuran dimer, kynapcin-24 (**83**), was later isolated from *P multiplex*. It inhibited PEP with an IC_50_ value of 1.14 µM [183]. Separation of a methanol extract of fruiting bodies of *P multiplex* collected in Korea afforded linoleic acid and oleic acid together with thelephoric acid [184]. Extracts of this mushroom were reported to have inhibitory effects on the proliferation of cancer cell lines [185], inhibitory activities (IC_50_ 10 μg/mL) against α-glucosidase [186] and DPPH radical scavenging activity [187]. Finally, it is worthy of note that polyozellin exhibits high important bioactivities, such as antioxidant [187], anti-carcinogenic [188] and inflammatory activities [189,190,191,192,193,194,195].

### 5.16. Ramaria botrytis (Pers.) Bourdot

*Ramaria botrytis* (Pers.) Bourdot, belonging to the family Ramariaceae, is a wild edible mushrooms which grows in mountains of eastern Asia, China, Thailand, Europe, and North America [4,58,196]. Zhou et al. reported the isolation of a novel ubiquitin-like antitumour protein which significantly inhibited the growth and induced apoptosis in A549 cells [196]. Bhanja and his collaborators isolated two water-insoluble glucans from the fruiting bodies of *R. botrytis* collected in India. One glucan was composed of (1→3)-linked α-d-glucopyranosyl residues and the other one was a β-d-glucan with a backbone of four (1→3)-linked β-d-glucopyranosyl units, with one single unit β-d-glucopyranosyl branch substituted at *O*-6 position [197]. A glucan consisting of (1→6)-linked-β-d glucopyranosyl residues as backbone, branched at *O*-3 position with a (1→3)-linked-β-d-glucopyranosyl unit and a non-reducing end β-d-glucopyranosyl residue has been purified by the same research group. This glucan showed immunostimulating activity on RAW 264.7, a murine macrophage cell line, by nitric oxide production [198]. Moreover, polysaccharides from *R. botrytis* showed potent antioxidant activities [199]. Fresh fruiting bodies of the mushroom collected in Japan have been reported to contain (2*S*,2′*R*,3*R*,4*E*,8*E*)-*N*-2′-hydroxyoctadecanoyl-2-amino-9-methyl-4,8-heptadecadiene-1,3-diol, 5α,6α-epoxy-3β-hydroxy-(22*E*)-ergosta-8(14),22-dien-7-one, ergosterol peroxide, cerevisterol and 9α-hydroxycerevisterol [200]. The in vitro antioxidant and antimicrobial potentials of extracts of *R. botrytis* were investigated by several research groups [26,201,202,203,204].

### 5.17. Rugiboletus extremiorientalis (Lj.N. Vassiljeva) G. Wu and Zhu L. Yang

*Rugiboletus extremiorientalis* (Lj.N. Vassiljeva) G. Wu & Zhu L. Yang [family Boletaceae, formerly named *Leccinum extremiorientale* (Lj.N. Vassiljeva) Singer] is an edible mushroom growing in northern temperate regions, especially in China, Laos and Thailand [4,58,205]. Leccinine A (**84**) and pyrrolezanthine (**85**) (see structures in Figure 16), were initially isolated from the mature fruiting bodies collected in Japan and showed protective activity against endoplasmic reticulum stress-dependent cell death [205]. Ito et al. isolated (8*E*,12*Z*)-10,11-dihydroxyoctadeca-8,12-dienoic acid and leccinine A, reporting their growth regulatory activity against lettuce [206]. Subsequently, the new pyrrole alkaloid 2-[2-formyl-5-(methoxymethyl)-1*H*-pyrrol-1-yl]acetic acid (**86**), together with 4-[2-formyl-5-(methoxymethyl)-1*H*-pyrrol-1-yl]butanoic acid and 4-[2-formyl-5-(hydroxymethyl)-1*H*-pyrrol-1-yl] butanoic acid were isolated from an ethyl acetate extract and exhibited poor cytotoxicity against K562, BEL7702, and SGC7901 cell lines with IC_50_ values higher than 40 µM [207]. The possible antioxidant and antimicrobial activities of secondary metabolites from *R. extremiorientalis* have not been examined so far.

### 5.18. Russula virescens (Schaeff.) Fr.

*Russula virescens* (Schaeff.) Fr., is a wild mushroom with a delicious taste, belonging to the family Russulaceae. It grows in nature on the roots of pine trees throughout China, Thailand, Lao, Nepal, and Europe [4,58]. The mushroom has long been used as a folk remedy in the traditional Chinese medicine [208]. Zhu et al. purified a novel laccase from *R. virescens* and then studied its dye decolorizing properties [209]. A water-insoluble linear (1→3)-β-d-glucan from the fresh fruiting bodies was isolated by the Sun’s group and did not exhibit antitumor activity, however, the sulfation of the native (1→3)-β-d-glucan improved the antitumor activity [210]. The extraction and purification of two novel water-soluble polysaccharides from fresh fruiting bodies of *R. virescens* were reported by the same research group. They revealed an interesting antioxidant properties [208]. Sun et al. [211] also isolated a water-soluble polysaccharide from the fruiting bodies of *R. virescens*, which had a backbone consisting of (1→6)-linked-α-d-galactopyranosyl and (1→2,6)-linked-α-d-galactopyranosyl residues that terminated in a single non-reducing terminal (1→)-α-d-mannopyranosyl residue at the *O*-2 position of each (1→2,6)-linked-α-d-galactopyranosyl residues along the main chain in the ratio of 1:1:1. The polysaccharide exhibited a significant scavenging effects of hydroxyl radicals in vitro. Canthin-6-one, 5α,8α-epidioxy-(22*E*,24*R*)-ergosta-6,22-dien-3β-ol, (22*E*,24*R*)-ergosta-5,7,22-trien-3β-ol, (22*E*,24*R*)-ergosta-7,22-dien-3β,5α,6β-triol, thioacetic anhydride, maleic acid, d-allitol and ribosidoadenine are secondary metabolites isolated from *R. virescens* [212]. Studies on the antioxidant activity of *R. virescens* revealed that this mushroom can be considered as an accessible source of natural antioxidants [204,206,213,214].

### 5.19. Sarcodon imbricatus (L.) P. Karst

*Sarcodon imbricatus* (L.) P. Karst, belonging to the family Bankeraceae, is an edible fungus occurring in China, Thailand, and Turkey [4,58,215]. It is widely used in Asian medicine [216]. An investigation on a polysaccharide-enriched extract of *S. imbricatus* revealed that it stimulates the immune response in CTX-induced immunosuppressed mice via modulation of oxidative pathways [216]. An extract of *S. imbricatus* exhibited the growth of gram-negative and gram-positive bacteria [22,217]. A Portugal research group reported that methanolic extracts of the mushroom showed potent antioxidant activity and antimicrobial activity against *Bacillus cereus* and *Cryptococcus neoformans* [218,219]. A new *p*-terphenyl, 2′,3′-diacetoxy-4,5,5′,6′,4″,5″-hexahydroxy-*p*-terphenyl (**87**), together with *p*-hydroxybenzoic acid, Bl-V (**88**), 2′,3′-diacetoxy-3,4,5′,6′,4″-pentahydroxy-*p*-terphenyl, cerebroside E (**89**) (see structures **87**−**89** in Figure 17), nicotinic acid, 4-allylcatechol, uracil, ethyl β-d-glucopyranoside, propanetriol, uridine, adenosine and d-allitol were isolated from the fruiting bodies [220]. In addition to ergosterol and ergosterol peroxide, *p*-hydroxybenzoic acid, protocatechuic acid, syringic acid, octanoic acid, decanoic acid, dodecanoic acid, tridecanoic acid, tetradecanoic acid, pentadecanoic acid, hexadecanoic acid, heptadecanoic acid, octadecanoic acid, eicosanoic acid, docosanoic acid, 9-tetradecenoic acid, 7-hexadecenoic acid, (*E*)-9-octadecenoic acid, (9*Z*)-octadecenoic acid, (13*Z*)-docosenoic acid, (9*Z*,12*Z*,15*Z*)-octadecatrienoic acid, 1-eicosenoic acid, (5*Z*,8*Z*,11*Z*,14*Z*)-eicosatetraenoic acid, methyl palmitate, methyl oleate, methyl linoleate and linolenic acid are the phenolic and fatty acids and esters isolated from this mushroom [221,222]. Polysaccharides isolated from *S. imbricatus* have demonstrated to possess antibacterial [223], anti-myelosuppressive [224], and immunomodulatory activities [225,226]. Fruiting bodies and/or mycelial cultures have been reported to possess antioxidant [203,221], antimicrobial [132], and antifatigue activities [227].

### 5.20. Termitomyces albuminosus (Berk.) R. Heim

*Termitomyces albuminosus* (Berk.) R. Heim, belonging to the family Lyophyllaceae is a very well-known wild edible mushroom, which is commonly distributed in Asia in China, Indonesia, Malaysia, and Singapore [4,228]. It cannot be cultivated, because a symbiotic relationship with termites is necessary [229,230]. The mushroom has been reported to contain water-soluble polysaccharides with a great variety of biological activities, including antioxidant, anti-inflammatory, hepatoprotective, hypolipidemic activities [230,231,232,233,234]. In addition, *T. albuminosus* has been reported to contain many other bioactive components, such as chitin-glucan complex, alkaline protease, saponins, melanin, lipids and ergosterol; some of which possess analgesic and anti-inflammatory activities [235,236,237,238]. Mau et al. studied a methanolic extract of *T. albuminosus* mycelia, reporting an interesting reducing power, scavenging activity and chelating effects of ferrous ions. [239]. Qi, et al. described the chemical structures of six novel cerebrosides, named termitomycesphins A–F (see structures **90**–**95** in Figure 18), together with known cerebroside **96**, and reported their neuritogenic activities [240,241]. Other two new cerebrosides, named termitomycesphins G (**97**) and H (**98**) were later isolated from this mushroom by the same research group [242]. Four new selinane-type sesquiterpenoids, named teucdiol C-F (see structures **99**–**102** in Figure 18), together with the known compounds teucdiol B (**103**) and epi-guaidiol A (**104**) were isolated by from a fermentation broth of *T. albuminosus* [243]; *epi*-guaidiol A (**104**) showed potent anti-acetylcholinesterase activity in a dose-dependent manner [243].

### 5.21. Termitomyces eurhizus (Berk.) R. Heim

*Termitomyces eurhizus* (Berk.) R. Heim, belonging to the family Lyophyllaceae, is a wild edible mushroom, which grows in association with termites in China, India, Myanmar, Malaysia, Nepal and Thailand [4,58,228,244]. Two water-soluble polysaccharides, whose structures were established to be (1→3)-d-Glc*p* and (1→6)-d-Glc*p*, and (1→6)-d-Glc*p* were isolated from a hot aqueous extract of fruiting bodies [244]. On the other hand, a water-insoluble (1→3)-β-d-glucan was isolated from a hot alkaline extract of the mushroom collected in India [245]. The biological activity of a water-soluble polysaccharide-rich fraction of *T. eurrhizus* was investigated by an Indian research group. The fraction revealed healing properties against indomethacin-induced stomach ulceration in mice [246]. Pharmacological studies on mushroom polysaccharides have highlighted other biological properties such as anticarcinogenic, antimicrobial, antioxidant and anti-inflammatory activities etc. [21]; therefore, *T. eurhizus* deserves further in-depth pharmacological investigations.

### 5.22. Termitomyces heimii Natarajan 

*Termitomyces heimii* Natarajan, (family-Lyophyllaceae) is a wild edible mushroom which grows in nature in symbiosis with termites in China, Malaysia, Thailand, and India [4,58,228,247]. Manna et al. reported the structure of a water-soluble β-glucan from this mushroom, together with its antioxidant activity [248]. The polysaccharide consisted of a backbone chain of two (1→6)-β-d-glucopyranosyl residues, one of which was branched at the *O*-3 position with a side chain consisting of two (1→3)-β-d-glucopyranosyl units and one terminal β-d-glucopyranosyl residue. The lipid content of *T. heimii* was analyzed by Abd Malek’s group who identified ergosterol and linoleic acid as the major components, and tetracosane, methyl palmitate, ethyl palmitate, methyl linoleate, ethyl linoleate, ethyl oleate, ethyl eicosanoate, ethyl tetracosanoate, ebericol, lanosterol, palmitic acid, oleic acid, stearic acid, neoergosterol, ergosta-5,8-dien-3-ol, ergosta-5,8(14)-dien-3-ol, 7-ergostenol, brassicasterol, γ-ergostenol, myristic acid, linoleic acid, benzaldehyde, 4-hydroxybenzaldehyde, benzeneacetamide, cinnamic acid and nicotinamide as the minor components [249]. A polyphenol-rich fraction of *T. heimii*, collected in West Bengal, showed potent antioxidant activity [247].

### 5.23. Termitomyces microcarpus (Berk. and Broome) R. Heim

*Termitomyces microcarpus* (Berk. & Broome) R. Heim (family Lyophyllaceae) is an edible mushroom which generally grows on termite material in China, Malaysia, Philippines, Thailand, India, and Nigeria [4,58,228,250,251]. Different α- and β-glucans were isolated from *T. microcarpus* and the repeating units of the new polysaccharides were identified by means of NMR studies and chemical investigations [252,253,254]. Dimethylincisterol, 5α,8α-epidioxy-(22*E*,24*R*)-ergosta-6,9(11),22-trien-3β-ol, 5α,8α-epidioxy-(22*E*,24*R*)-ergosta-6,22-dien-3β-ol, 5α,6α-epoxy-(22*E*,24*R*)-ergosta-8(14),22-diene-3β,7α-diol, (22*E*,24*R*)-ergosta-7,22-diene-3β,5α,6β-triol, and betulinic acid were isolated by Njue et al., who also reported their cytotoxic activities [255]. Nakalembe and Kabasa studied the antimicrobial activity and the bioactive compounds from *T. microcarpus* collected in Uganda, using GC-MS [256]; an interesting antimicrobial activity, especially against *S. aureus* and *P. aeruginosa*, was determined. In other studies, a mushroom extract displayed significant antioxidant and free radical scavenging activities [37,257,258,259].

### 5.24. Thelephora ganbajun M. Zang

*Thelephora ganbajun* M. Zang, belonging to the family Thelephoraceae, is one of the most favorite edible mushrooms. It widely grows in symbiosis with pine trees in China and the Greater Mekong region [260], where it is highly prized for its unique taste and flavor [260,261]. A novel ribonuclease, showing potent inhibitory activity toward HIV-1 reverse transcriptase, was isolated from dried fruiting bodies of the mushroom by Wang and Ng [262]. Two new polysaccharide fractions isolated from the fruiting bodies were characterized by Gong’s group [263]. They exhibited strong inhibitory effects on HeLa cells and moderate inhibitory effect on α-amylase and α-glucosidase. Separation of an EtOAc-partitioned MeOH extract of *T. ganbajun* fruiting bodies collected in China afforded, in addition to 3-*O*-methylatromentin, five new poly(phenylacetyloxy)-substituted 1,1′:4′,1″-terphenyl derivatives, called ganbajunins A–E (see structures **105**–**109** in Figure 19) [264]. Subsequently, ganbajunin F and G (see structures **110**–**111** in Figure 19), together with cycloleucomelone were isolated from fresh fruiting bodies by the same research group [265]. The extracts obtained under optimized conditions by an ultrasonic-assisted extraction procedure, possessed significant antiproliferative activities towards human lung and liver cancer cells [266]. Moreover, ganbajunins A–C (**105**–**107**) and 3-*O*-methylatromentin possessed potent lipid peroxidation inhibitory activity, SOD activity in rat liver homogenate, and DPPH radical scavenging activity [261,267,268].

### 5.25. Volvariella Bombycina (Schaeff.) Singer

*Volvariella bombycina* (Schaeff.) Singer is a wild edible mushroom belonging to the family Pluteaceae which grows in Asia in China and Thailand [4,58,269]. Das et al. [270] isolated a water-soluble polysaccharide from the hot aqueous extract of the mushroom collected in India. The repeating unit was identified as a →6)-β-d-Glc*p*-(1→6)-α-d-Man*p*-(1→6)-α-d-Glc*p*-(1→ backbone to which an α-d-galactosyl unit was attached. A novel compound, named isodeoxyhelicobasidin (structure **112** in Figure 20), was isolated by a Korean research group from a culture broth of *V. bombycine*. Compound **112** was reported to possess human neutrophil elastase (HNE) activity with an IC_50_ value of 9.0 µm and antibacterial activity against several gram-positive bacteria, including *S. aureus* 503, methicillin-resistant *S. aureus* CCARM 3167 (MRSA), quinolone-resistant *S. aureus* CCARM 3505 (QRSA), *Bacillus subtilis* 1021, *Staphylococcus epidermidis* 3958 and *Streptococcus mutans* 3065, with MIC values in the range of 3.1–12.4 µg/mL [271]. Ergosta-4,6,8(14),22-tetraene-3-one (**113**), ergosterol peroxide, indole-3-carboxaldehyde (**114**), and indazole (**115**) were later isolated from a culture broth of *V. bombycine*. Compound **113** showed inhibitory activity on melanogenesis with an IC_50_ = 80.9 µM and cytotoxic activity with an LD_50_ value of 50.6 μM [272]. Moreover, a *V. bombycina* extract showed a moderate antioxidant activity [269,273].

## 6. Conclusions

Southeast Asia is one of the biodiversity hot-spots in the world and has an outstanding rate of species discovery. In fact, hundreds of new species are described annually. However, regional biological resources are currently threatened by climatic changes and human activity-related factors such as the high rate of mining in the tropics, the construction of a great number of hydropower dams, and an indiscriminate consumption of plants in traditional medicines [274,275,276]. Therefore, access to biodiversity resources of Southeast Asia must be done paying great attention to their conservation or renovation. In this context, mushrooms play important roles in different ecosystems; however, they are often obtained in artificial cultures, thus avoiding the collection in the wild.

Although the variety of higher mushroom (Basidiomycetes) growing in Southeast Asia is calculated to be very high, only few scientific mycological investigations have been conducted, and most species growing in countries such as Myanmar, Laos, and Cambodia, have not been identified so far.

We believe that this review clearly demonstrates that edible mushrooms are a rich source of various bioactive substances having antimicrobial, antioxidant, anti-inflammatory, anti-proliferative, cytotoxic, anti-HIV, anti-diabetic properties, among other ones. Therefore, edible mushrooms must be considered not only culinary delicacies but also functional foods and, in some cases, even therapeutic agents. Of course, mushroom edibility is a proof of their non-acute toxicity. Therefore, edible mushrooms containing bioactive compounds can have high potential as sources of medicinal remedies.

## Figures and Tables

**Table 1:** Biological/pharmacological activities of extracts/compounds isolated from selected wild edible mushrooms growing in Southeast Asia countries.

Mushroom Species	Metabolites with Antioxidant Activity	Metabolites with Antimicrobial Activity	Metabolites with Cytotoxic and/or Antiproliferative Activity	Metabolites with Other Biological Activities
*Agaricus silvaticus* Schaeff. (Agaricaceae)	Extract [64,65]	-	-	-
*Ampulloclitocybe clavipes* (Pers.) Redhead, Lutzoni, Moncalvo & Vilgalys (Hygrophoraceae)	-	Clavilactone A-C (**4**−**6**) [66]	Clavipines A (**9**) [70], Clavilactone H (**16**) [71]	Clavilactone B (**5**) (inhibitory activity of the growth of *Lepidum sativum*) [66]). Clavilactones A, B, D (**4,5,8**) (tyrosine kinases inhibitory activity [67,69]). Fatty acid derivatives (aldehyde dehydrogenase inhibitory activity [65])
*Butyriboletus roseoflavus* (M. Zang & H.B. Li) D. Arora & J.L. Frank (Boletaceae)	Polysaccharide [72]	-	Polysaccharide [73,74,75], Hemagglutinin [76]	Polysaccharide (immunoregulatory activity [73,74,75]). Hemagglutinin (HIV-1 reverse transcriptase inhibitory activity [76])
*Cantharellus cibarius* Fr. (Cantharellaceae)	Polysaccharide [85,86]	Extract [92]	Polysaccharide [80,82]	Polysaccharide (immunomodulatory [81,86] and neuroprotective activities [85])
*Craterellus cornucopioides* (L.Fr.) Pers. (Cantharellaceae)	Polysaccharide [97], Extract [91,93,105,106,108]	Extract [105]	Craterellin C (**20**) [98], Extract [93,105]	Polysaccharide [94,96]. Extract (antimutagenic effects [105], antihyperglycemic [106] and anti-inflammatory activities [107,108])
*Craterellus odoratus* (Schwein.) Fr. (Cantharellaceae)	-	-	Calbistrin C [112]	Craterellin A (**26**) (inhibitory activities against human 11β-HSD2 [111]). 5-Hydroxymethyl-2-hydroxy-4-methoxy-phenylethanone (inhibitory activity against human 11β-HSD1 [109])
*Fistulina hepatica* (Schaeff.) (Fistulinaceae)	Extract [115,118,119]	Cinnatriacetins A (**37**) and B (**38**) [117]. Extract [22,116,125]	-	-
*Hydnum repandum* L. (Cantharellaceae)	Extract [92,115,127,133]	Extract [92,126,131,132]	Repandiol (**39**) [129], Extract [93]	-
*Laccaria amethystea* (Bull.) Murrill (Hydnangiaceae)	Extract [106]	Laccaridiones A (**41**) and B (**42**) [134]	Laccaridiones B (**42**) [134]	Extract (antihyperglycemic activity [106])
*Lactarius hatsudake* Nobuj. Tanaka (Russulaceae)	-	-	-	Ergosterol peroxide, 5α,8α-epidioxy-(24*S*)-ergosta -6-en-3β-ol (antiphospholipase A_2_ activity [139] and anti-HIV activity [140])
*Lepista sordida* (Schumach.) Singer (Tricholomataceae)	Polysaccharides [148]	Lepistal (**48**), lepistol (**49**) [149]	Polysaccharides [146,147], lepistal (**48**), lepistol (**49**) [149]	Polysaccharide (immunoregulatory activity [145]). Compounds **56**−**59** (plant growth inhibitory activity [155])
*Lycoperdon pyriforme* Schaeff. (Agaricaceae)	Extract [161]	Compounds **63** and **64** [158]. Extract [160]	Compound **65** [158]	Compounds **63** and **64** (nematicidal activity [158])
*Neolentinus lepideus* (Fr.) Redhead & Ginns (Polyporaceae)	Compound **68** [166], Extract [168]	-	-	Lepidepyrone (**66**) (inhibitory effects on hyaluronidase [162). Compounds **67** and **68** (NO inhibitory activity [166]) Compounds **69** and **70** (tyrosinase inhibitory activity [167]). Polysaccharide (immunomodulating activity [169]). Extract (antityrosinase [168], antihyperlipidemic [163] and immunomodulatory activities [170])
*Phlebopus portentosus* (Berk. & Broome) Boedijn (Boletinellaceae)	Extract [173]	-	-	Extract (tyrosinase and hyperglycaemic moderate inhibitory activities [173]). Compound **74** (neuroprotective activity [174])
*Polyozellus multiplex* (Underw.) Murrill (Thelephoraceae)	Polyozellin (**75**) and extract [189]	Extract [188]	Polyozellin (**75**) [179,190]. Extract [187,190]	Polyozellin (**75**) (prolyl endopeptidase (PEP) inhibitory activity [176,177], β-secretase (BACE1) inhibitory activities [175], neuroprotective effect [187] and anti-inflammatory activities [189,190,191,192,193,194,195]). Thelephoric acid (**76**) (PEP inhibitory activity [178], β-secretase (BACE1) inhibitory activity [175] and neuroprotective effect [175]). Kynapcin-9 (**77**) (PEP inhibitory activity) [178]. Kynapcin-12 (**78**) (PEP inhibitory [179], prolyl oligopeptidase (POP) inhibitory [180] and β-secretase (BACE1) inhibitory activities [175]). Polyozellic acid (**79**) (antiangiogenesis [181], β-secretase (BACE1) inhibitory activities [175] and neuroprotective effects [175]), compound **80** (antiangiogenesis activity [181]). Kynapcin-13 (**81**) and -28 (**82**) (PEP inhibitory activity [182]), Kynapcin-24 (**83**) (PEP inhibitory activity [183]).
*Ramaria botrytis* (Pers.) Bourdot (Ramariaceae)	Polysaccharide [199], Extract [202,203,204]	-	A novel ubiquitin-like protein [196]	Glucan (immunostimulating activity) [198]
*Rugiboletus extremiorientalis* (Lj.N. Vassiljeva) G. Wu & Zhu L. Yang	-	-	-	Leccinine A (**84**) (protective activity against endoplasmic reticulum stress-dependent cell death [205] and plant growth regulatory activity [206]). (8*E*,12*Z*)-10,11-dihydroxyoctadeca-8,12-dienoic acid (plant growth regulatory activity [206]).
*Russula virescens* (Schaeff.) Fr. (Russulaceae)	Polysaccharide [208,211], Extract [203,213,214]	-	-	-
*Sarcodon imbricatus* (L.) P. Karst (Bankeraceae)	Extract [203,219,221]	Extract [22,132,217,218], Polysaccharide [223]	-	Extract (immunomodulatory [216] and antifatigue activities [227]). Polysaccharide (immunoenhancement [225,226] and anti-myelosuppressive activities [224]).
*Termitomyces albuminosus* (Berk.) R. Heim (Lyophyllaceae)	Polysaccharide [230,232,233,234], Extract [239]	-	-	Polysaccharide (anti-inflammatory [234] and hepatoprotective effects [232,234]). Extract (analgesic and anti-inflammatory activities [236]). Termitomycesphins A-F (**90**-**95**) (neuritogenic activity [240,241]). Termitomycesphins G (**97**) and H (**98**) (neuritogenic activity [242]). *epi*-Guaidiol A (**104**) (anti-acetylcholinesterase activity [243]).
*Termitomyces eurhizus* (Berk.) R. Heim (Lyophyllaceae)	-	-	-	Extract (anti-ulcerogenic activity) [245]
*Termitomyces heimii* Natarajan (Lyophyllaceae)	Extract [247] Polysaccharide [248]	-	-	-
*Termitomyces microcarpus* (Berk. & Broome) R. Hein (Lyophyllaceae)	Extract [36,257,258,259]	Extract [257]	Dimethylincisterol; 5α,8α-epidioxy-(22*E*,24*R*)-ergosta-6,22-dien-3β-ol [255]	-
*Thelephora ganbajun* M. Zang (Thelephoraceae)	Ganbajunins A-B (**105**−**106**) [261,267,268]. Ganbajunin C (**107**); 3-*O*-methylatromentin [267,268]	-	Polysaccharide [263], Extract [266]	Ribonuclease (inhibitory activity toward HIV-1 reverse transcriptase) [262]. Polysaccharides (antidiabetic activity) [263]
*Volvariella bombycina* (Schaeff.) Singer (Pluteaceae)	Extract [269,273]	Isodeoxyhelicobasidin [271]	Compound **113** [272]	Isodeoxyhelicobasidin (human neutrophil elastase (NHE) activity [271]). Compound **113** (inhibitory effects on melanogenesis [272]).

## References

[1] Hawksworth D.L. (2001). The magnitude of fungal diversity: The 1.5 million species estimate revisited. Mycol. Res..

[2] Hawksworth D.L., Lücking R. (2017). Fungal diversity revisited: 2.2 to 3.8 million species. Microbiol. Spectr..

[3] He M.-Q., Zhao R.-L., Hyde K.D., Begerow D., Kemler M., Yurkov A., McKenzie E.H.C., Raspé O., Kakishima M., Sánchez-Ramírez S. (2019). Notes, outline and divergence times of Basidiomycota. Fungal Divers..

[4] Boa E. (2004). Wild Edible Fungi: A Global Overview of Their Use and Importance to People.

[5] Kalač P. (2009). Chemical composition and nutritional value of European species of wild growing mushrooms: A review. Food Chem..

[6] Srikram A., Supapvanich S. (2016). Proximate compositions and bioactive compounds of edible wild and cultivated mushrooms from Northeast Thailand. Agric. Nat. Resour..

[7] Díez V.A., Alvarez A. (2001). Compositional and nutritional studies on two wild edible mushrooms from Northwest Spain. Food Chem..

[8] Günç Ergönül P., Akata I., Kalyoncu F., Ergönül B. (2013). Fatty acid compositions of six wild edible mushroom species. Sci. World J..

[9] Sarikurkcu C., Copur M., Yildiz D., Akata I. (2011). Metal concentration of wild edible mushrooms in Soguksu National Park in Turkey. Food Chem..

[10] Surinrut P., Julshamn K., Rein Njaa L. (1987). Protein, amino acids and some major and trace elements in Thai and Norwegian mushrooms. Plant. Food Hum. Nutr..

[11] Xu X., Yan H., Chen J., Zhang X. (2011). Bioactive proteins from mushrooms. Biotechnol. Adv..

[12] Zhou R., Liu Z.K., Zhang Y.N., Wong J.H., Ng T.B., Liu F. (2019). Research progress of bioactive proteins from the edible and medicinal mushrooms. Curr. Protein Pept. Sci..

[13] Wang X.M., Zhang J., Wu L.H., Zhao Y.L., Li T., Li J.Q., Wang Y.Z., Liu H.G. (2014). A mini-review of chemical composition and nutritional value of edible wild-grown mushroom from China. Food Chem..

[14] Falandysz J. (2017). Mercury accumulation of three *Lactarius* mushroom species. Food Chem..

[15] Mironczuk-Chodakowska I., Socha K., Zujko M.E., Terlikowska K.M., Borawska M.H., Witkowska A.M. (2019). Copper, manganese, selenium and zinc in wild-growing edible mushrooms from the Eastern territory of “green lungs of Poland”: Nutritional and toxicological implications. Int. J. Environ. Res. Public Health..

[16] Rashid M.H., Rahman M.M., Correll R., Naidu R. (2018). Arsenic and other elemental concentrations in mushrooms from Bangladesh: Health risks. Int. J. Environ. Res. Public Health..

[17] Fang Y.-Z., Yang S., Wu G. (2002). Free radicals, antioxidants, and nutrition. Nutrition.

[18] Kozarski M., Klaus A., Jakovljevic D., Todorovic N., Vunduk J., Petrović P., Niksic M., Vrvic M.M., van Griensven L. (2015). Antioxidants of edible mushrooms. Molecules.

[19] Elkhateeb W.A., Daba G.M., Thomas P.W., Wen T.-C. (2019). Medicinal mushrooms as a new source of natural therapeutic bioactive compounds. Egypt. Pharm. J..

[20] Hsieh H.-M., Ju Y.-M. (2018). Medicinal components in *Termitomyces* mushrooms. Appl. Microbiol. Biotechnol..

[21] Friedman M. (2016). Mushroom polysaccharides: Chemistry and antiobesity, antidiabetes, anticancer, and antibiotic properties in cells, rodents, and humans. Foods.

[22] Alves M.J., Ferreira I.C., Martins A., Pintado M. (2012). Antimicrobial activity of wild mushroom extracts against clinical isolates resistant to different antibiotics. J. Appl. Microbiol..

[23] Gebreyohannes G., Nyerere A., Bii C., Berhe Sbhatu D. (2019). Determination of antimicrobial activity of extracts of indigenous wild mushrooms against pathogenic organisms. Evid. Based Complementary Altern. Med..

[24] Ramesh C., Pattar M.G. (2010). Antimicrobial properties, antioxidant activity and bioactive compounds from six wild edible mushrooms of Western Ghats of Karnataka, India. Pharmacogn. Res..

[25] Venturini M., Rivera C., Gonzalez C., Blanco D. (2008). Antimicrobial activity of extracts of edible wild and cultivated mushrooms against foodborne bacterial strains. J. Food Prot..

[26] Barros L., Venturini B.A., Baptista P., Estevinho L.M., Ferreira I.C.F.R. (2008). Chemical composition and biological properties of Portuguese wild mushrooms: A comprehensive study. J. Agric. Food Chem..

[27] Goh D.P.S., Orum A.M. (2019). Southeast Asia. The Wiley Blackwell Encyclopedia of Urban and Regional Studies.

[28] Gasparatos A., Subramanian S.M., Elliott W., Braimoh A.K. Unraveling the Drivers of Southeast Asia’s Biodiversity Loss. https://ourworld.unu.edu/en/unraveling-the-drivers-of-southeast-asia%e2%80%99s-biodiversity-loss.

[29] WWF New Species Discoveries in the Greater Mekong. https://wwf.panda.org/?222513/New-species-discoveries-in-the-Greater-Mekong.

[30] Hyde K.D., Norphanphoun C., Chen J., Dissanayake A.J., Doilom M., Hongsanan S., Jayawardena R.S., Jeewon R., Perera R.H., Thongbai B. (2018). Thailand’s amazing diversity: Up to 96% of fungi in northern Thailand may be novel. Fungal Divers..

[31] Valko M., Izakovic M., Mazur M., Rhodes C.J., Telser J. (2004). Role of oxygen radicals in DNA damage and cancer incidence. Mol. Cell. Biochem..

[32] Valko M., Rhodes C.J., Moncol J., Izakovic M., Mazur M. (2006). Free radicals, metals and antioxidants in oxidative stress-induced cancer. Chem. Biol. Interact..

[33] Winterbourn C.C. (1993). Superoxide as an intracellular radical sink. Free Radic. Biol. Med..

[34] Diplock A.T., Charleux J.L., Crozier-Willi G., Kok F.J., Rice-Evans C., Roberfroid M., Stahl W., Viña-Ribes J. (1998). Functional food science and defence against reactive oxidative species. Br. J. Nutr..

[35] Valko M., Leibfritz D., Moncol J., Cronin M.T.D., Mazur M., Telser J. (2007). Free radicals and antioxidants in normal physiological functions and human disease. Int. J. Biochem. Cell Biol..

[36] Simic M.G. (1988). Mechanisms of inhibition of free-radical processes in mutagenesis and carcinogenesis. Mutat. Res..

[37] Bouayed J., Bohn T. (2010). Exogenous antioxidants—double-edged swords in cellular redox state: Health beneficial effects at physiologic doses versus deleterious effects at high doses. Oxid. Med. Cell. Longev..

[38] Shahidi F. (2000). Antioxidants in food and food antioxidants. Nahrung.

[39] Kim M.-Y., Seguin P., Ahn J.-K., Kim J.-J., Chun S.-C., Kim E.-H., Seo S.-H., Kang E.-Y., Kim S.-L., Park Y.-J. (2008). Phenolic compound concentration and antioxidant activities of edible and medicinal mushrooms from Korea. J. Agric. Food Chem..

[40] Kumari D., Reddy M.S., Upadhyay R.C. (2011). Nutritional composition and antioxidant activities of 18 different wild *Cantharellus* mushrooms of Northwestern Himalayas. Food Sci. Technol. Int..

[41] Puttaraju N.G., Venkateshaiah S.U., Dharmesh S.M., Urs S.M.N., Somasundaram R. (2006). Antioxidant activity of indigenous edible mushrooms. J. Agric. Food Chem..

[42] Woodford N., Livermore D.M. (2009). Infections caused by gram-positive bacteria: A review of the global challenge. J. Infect..

[43] Prestinaci F., Pezzotti P., Pantosti A. (2015). Antimicrobial resistance: A global multifaceted phenomenon. Pathog. Glob. Health.

[44] Vacca P., Fazio C., Neri A., Ambrosio L., Palmieri A., Stefanelli P. (2018). *Neisseria meningitidis* antimicrobial resistance in Italy, 2006 to 2016. Antimicrob. Agents Chemother..

[45] Watkins R.R., Holubar M., David M.Z. (2019). Antimicrobial resistance in methicillin-resistant *Staphylococcus aureus* to newer antimicrobial agents. Antimicrob. Agents Chemother..

[46] O’Donnell F., Smyth T.J.P., Ramachandran V.N., Smyth W.F. (2010). A study of the antimicrobial activity of selected synthetic and naturally occurring quinolines. Int. J. Antimicrob. Agents..

[47] Smyth T., Ramachandran V.N., Smyth W.F. (2009). A study of the antimicrobial activity of selected naturally occurring and synthetic coumarins. Int. J. Antimicrob. Agents..

[48] Barbieri R., Coppo E., Marchese A., Daglia M., Sobarzo-Sánchez E., Nabavi S.F., Nabavi S.M. (2017). Phytochemicals for human disease: An update on plant-derived compounds antibacterial activity. Microbiol. Res..

[49] Khameneh B., Iranshahy M., Soheili V., Fazly Bazzaz B.S. (2019). Review on plant antimicrobials: A mechanistic viewpoint. Antimicrob. Resist. Infect. Control..

[50] Tambekar D.H., Sonar T.P., Khodke M.V., Khante B.S. (2006). The novel antibacterials from two edible mushrooms: *Agaricus bisporus* and *Pleurotus sajor caju*. Int. J. Pharmacol..

[51] Alves M.J., Ferreira I.C.F.R., Dias J., Teixeira V., Martins A., Pintado M. (2012). A review on antimicrobial activity of mushroom (Basidiomycetes) extracts and isolated compounds. Planta Med..

[52] Hatab S., Athanasio R., Holley R., Rodas-Gonzalez A., Narvaez-Bravo C. (2016). Survival and reduction of shiga toxin-producing *Escherichia coli* in a fresh cold-pressed juice treated with antimicrobial plant extracts. J. Food Sci..

[53] Bondi M., Lauková A., de Niederhausern S., Messi P., Papadopoulou C. (2017). Natural preservatives to improve food quality and safety. J. Food Qual..

[54] Olatunde O.O., Benjakul S. (2018). Natural preservatives for extending the shelf-life of seafood: A revisit. Compr. Rev. Food Sci. Food Saf..

[55] Shen H.S., Shao S., Chen J.C., Zhou T. (2017). Antimicrobials from mushrooms for assuring food safety. Compr. Rev. Food Sci. Food Saf..

[56] Burt S. (2004). Essential oils: Their antibacterial properties and potential applications in foods—A review. Int. J. Food Microbiol..

[57] Lodonjav M., Luo G., Zhou M., Duger R., Zhang G., Luo Y. (2014). Chemical components from an edible mushroom *Agaricus silvaticus*. Chin. J. Appl. Environ. Biol..

[58] Chandrasrikul A., Suwanarit P., Sangwanit U., Lumyong S., Payapanon A., Sanoamuang N., Pukahuta C., Petcharat V., Sardsud U., Duengkae K. (2011). Checklist of Mushrooms (Basidiomycetes) in Thailand.

[59] Boonyanuphap J., Hansawasdi C. (2011). Spatial distribution of beta glucan containing wild mushroom communities in subtropical dry forest, Thailand. Fungal Divers..

[60] Chilton W.S., Chien P.H. (1975). *N*-nitroamines of *Agaricus silvaticus*. Phytochemistry.

[61] Alston T.A., Porter D.J., Seitz S.P., Bright H.J. (1981). Oxidation of *N*-nitroethylenediamine, a GABA analog from *Agaricus silvaticus*, by GABA aminotransferase. Biochem. Biophys. Res. Commun..

[62] Nilsson L., Noori G., Bergman R., Kesler E., Sterner O. (1983). A novel synthesis of β-aminoalkylnitroamines. *rac*-β-nitroaminoalanine and *N*-nitroethylenediamine, two reported metabolites from *Agaricus silvaticus*. Acta Chem. Scand..

[63] Garrab M., Edziri H., El Mokni R., Mastouri M., Mabrouk H., Douki W. (2019). Phenolic composition, antioxidant and anticholinesterase properties of the three mushrooms *Agaricus silvaticus* Schaeff., *Hydnum rufescens* Pers. and *Meripilus giganteus* (Pers.) Karst. in Tunisia. S. Afr. J. Bot..

[64] Gąsecka M., Magdziak Z., Siwulski M., Mleczek M. (2018). Profile of phenolic and organic acids, antioxidant properties and ergosterol content in cultivated and wild growing species of *Agaricus*. Eur. Food Res. Technol..

[65] Kawagishi H., Miyazawa T., Kume H., Arimoto Y., Inakuma T. (2002). Aldehyde dehydrogenase inhibitors from the mushroom *Clitocybe clavipes*. J. Nat. Prod..

[66] Arnone A., Cardillo R., Meille S.V., Nasini G., Tolazzi M. (1994). Secondary mould metabolites. Part 47. Isolation and structure elucidation of clavilactones A–C new metabolites from the fungus *Clitocybe clavipes*. J. Chem. Soc. Perkin Trans. 1.

[67] Merlini L., Nasini G., Scaglioni L., Cassinelli G., Lanzi C. (2000). Structure elucidation of clavilactone D: An inhibitor of protein tyrosine kinases. Phytochemistry.

[68] Takao K.-i., Mori K., Kasuga K., Nanamiya R., Namba A., Fukushima Y., Nemoto R., Mogi T., Yasui H., Ogura A. (2018). Total synthesis of clavilactones. J. Org. Chem..

[69] Cassinelli G., Lanzi C., Pensa T., Gambetta R.A., Nasini G., Cuccuru G., Cassinis M., Pratesi G., Polizzi D., Tortoreto M. (2000). Clavilactones, a novel class of tyrosine kinase inhibitors of fungal origin. Biochem. Pharmacol..

[70] Sun Z., Zhu N., Zhou M., Huo X., Wu H., Tian Y., Yang J., Ma G., Yang Y.-L., Xu X. (2019). Clavipines A–C, antiproliferative meroterpenoids with a fused azepine skeleton from the basidiomycete *Clitocybe clavipes*. Org. Chem. Front..

[71] Sun Z., Xu X., Liang H., Xia X., Ma G., Shi L. (2019). Five new meroterpenoids from the fruiting bodies of the basidiomycete *Clitocybe clavipes* with cytotoxic activity. Molecules.

[72] Ding X., Hou Y.-L., Hou W.-R. (2012). Structure elucidation and antioxidant activity of a novel polysaccharide isolated from *Boletus speciosus* Forst. Int. J. Biol. Macromol..

[73] Su S., Wang M., Ding X., Hou Y., Tang J., Liu L., Dong M., Jing L. (2018). Protein chip of *Boletus speciosus* Frost polysaccharide revealed the molecular mechanism of antitumor and immunostimulatory activities on macrophages. Indian, J. Pharm. Sci..

[74] Hou Y., Ding X., Hou W., Song B., Wang T., Wang F., Li J., Zeng Y., Zhong J., Xu T. (2014). Pharmacological evaluation for anticancer and immune activities of a novel polysaccharide isolated from *Boletus speciosus* Frost. Mol. Med. Rep..

[75] Zhu H., Ding X., Hou Y., Li Y., Wang M. (2019). Structure elucidation and bioactivities of a new polysaccharide from Xiaojin *Boletus speciosus* Frost. Int. J. Biol. Macromol..

[76] Sun J., Ng T.-B., Wang H., Zhang G. (2014). A novel hemagglutinin with antiproliferative activity against tumor cells from the hallucinogenic mushroom *Boletus speciosus Biomed*. Res. Int..

[77] Eyssartier G., Stubbe D., Walleyn R., Verbeken A. (2009). New records of *Cantharellus* species (Basidiomycota, *Cantharellaceae*) from Malaysian dipterocarp rainforest. Fungal Divers..

[78] Jones E.B.G., Whalley A.J.S., Hywel-Jones N.L. (1994). A fungus foray to Chiang Mai market in Northern Thailand. Mycologist.

[79] Olariaga I., Moreno G., Manjón J.L., Salcedo I., Hofstetter V., Rodríguez D., Buyck B. (2017). *Cantharellus* (Cantharellales, Basidiomycota) revisited in Europe through a multigene phylogeny. Fungal Divers..

[80] Meng Y., Qu Y., Wu W., Chen L., Sun L., Tai G., Zhou Y., Cheng H. (2019). Galactan isolated from *Cantharellus cibarius* modulates antitumor immune response by converting tumor-associated macrophages toward M1-like phenotype. Carbohydr. Polym..

[81] Yang G., Qu Y., Meng Y., Wang Y., Song C., Cheng H., Li X., Sun L., Zhou Y. (2019). A novel linear 3-*O*-methylated galactan isolated from *Cantharellus cibarius* activates macrophages. Carbohydr. Polym..

[82] Nowacka-Jechalke N., Nowak R., Juda M., Malm A., Lemieszek M., Rzeski W., Kaczyński Z. (2018). New biological activity of the polysaccharide fraction from *Cantharellus cibarius* and its structural characterization. Food Chem..

[83] Villares A., García-Lafuente A., Guillamón E., Mateo-Vivaracho L. (2013). Separation and characterization of the structural features of macromolecular carbohydrates from wild edible mushrooms. Bioact. Carbohydr. Diet. Fibre..

[84] Nyman A.A.T., Aachmann F.L., Rise F., Ballance S., Samuelsen A.B.C. (2016). Structural characterization of a branched (1→6)-α-mannan and β-glucans isolated from the fruiting bodies of *Cantharellus cibarius*. Carbohydr. Polym..

[85] Lemieszek M.K., Nunes F.M., Cardoso C., Marques G., Rzeski W. (2018). Neuroprotective properties of *Cantharellus cibarius* polysaccharide fractions in different *in vitro* models of neurodegeneration. Carbohydr. Polym..

[86] Zhao D., Ding X., Hou Y., Hou W., Liu L., Xu T., Yang D. (2018). Structural characterization, immune regulation and antioxidant activity of a new heteropolysaccharide from *Cantharellus cibarius* Fr. Int. J. Mol. Med..

[87] Mittermeier V.K., Dunkel A., Hofmann T. (2018). Discovery of taste modulating octadecadien-12-ynoic acids in golden chanterelles (*Cantharellus cibarius*). Food Chem..

[88] Pang Z., Sterner O. (1991). Cibaric acid, a new fatty acid derivative formed enzymically in damaged fruit bodies of *Cantharellus cibarius* (chanterelle). J. Org. Chem..

[89] Hong S.S., Lee J.H., Jeong W., Kim N., Jin H.Z., Hwang B.Y., Lee H.-J., Lee S.-J., Jang D.S., Lee D. (2012). Acetylenic acid analogues from the edible mushroom chanterelle (*Cantharellus cibarius*) and their effects on the gene expression of peroxisome proliferator-activated receptor-gamma target genes. Bioorg. Med. Chem. Lett..

[90] Rangel-Castro J.I., Staffas A., Danell E. (2002). The ergocalciferol content of dried pigmented and albino *Cantharellus cibarius* fruit bodies. Mycol. Res..

[91] Palacios I., Lozano M., Moro C., D’Arrigo M., Rostagno M.A., Martínez J.A., García-Lafuente A., Guillamón E., Villares A. (2011). Antioxidant properties of phenolic compounds occurring in edible mushrooms. Food Chem..

[92] Ozen T., Darcan C., Aktop O., Turkekul I. (2011). Screening of antioxidant, antimicrobial activities and chemical contents of edible mushrooms wildly grown in the Black Sea region of Turkey. Comb. Chem. High. Throughput Screen..

[93] Vasdekis E.P., Karkabounas A., Giannakopoulos I., Savvas D., Lekka M.E. (2018). Screening of mushrooms bioactivity: Piceatannol was identified as a bioactive ingredient in the order Cantharellales. Eur. Food Res. Technol..

[94] Guo M.-Z., Meng M., Duan S.-Q., Feng C.-C., Wang C.-L. (2019). Structure characterization, physicochemical property and immunomodulatory activity on RAW264.7 cells of a novel triple-helix polysaccharide from *Craterellus cornucopioides*. Int. J. Biol. Macromol..

[95] Hall I.R., Stephenson S.L., Buchanan P.K., Yun W., Cole A.L.J. (2003). Edible and Poisonous Mushrooms of the World.

[96] Guo M.-Z., Meng M., Feng C.-C., Wang X., Wang C.-L. (2019). A novel polysaccharide obtained from *Craterellus cornucopioides* enhances immunomodulatory activity in immunosuppressive mice models via regulation of the TLR4-NF-κB pathway. Food Funct..

[97] Yang W.-W., Wang L.-M., Gong L.-L., Lu Y.-M., Pan W.-J., Wang Y., Zhang W.-N., Chen Y. (2018). Structural characterization and antioxidant activities of a novel polysaccharide fraction from the fruiting bodies of *Craterellus cornucopioides*. Int. J. Biol. Macromol..

[98] Guo H., Diao Q.-P., Hou D.-Y., Li Z.-H., Zhou Z.-Y., Feng T., Liu J.-K. (2017). Sesquiterpenoids from cultures of the edible mushroom *Craterellus cornucopioides*. Phytochem. Lett..

[99] Guo H., Diao Q.-P., Zhang B., Feng T. (2019). Two new illudane sesquiterpenoids and one new menthane monoterpene from cultures of *Craterellus cornucopioides*. J. Asian Nat. Prod. Res..

[100] Liu R., Zhou Z.-Y., Liu J.-K. (2010). Three new keto esters from cultures of the Basidiomycete *Craterellus cornucopioides*. Chin. J. Nat. Med..

[101] Magnus V., Laćan G., Iskrić S., Lewer P., Aplin R.T., Thaller V. (1989). Conversion of indole-3-ethanol to fatty acid esters in *Craterellus cornucopioides*. Phytochemistry.

[102] Magnus V., Laćan G., Aplin R.T., Thaller V. (1989). Glycerol tridehydrocrepenynate from the Basidiomycete *Craterellus cornucopioides*. Phytochemistry.

[103] Villares A., Mateo-Vivaracho L., García-Lafuente A., Guillamón E. (2014). Storage temperature and UV-irradiation influence on the ergosterol content in edible mushrooms. Food Chem..

[104] Watanabe F., Schwarz J., Takenaka S., Miyamoto E., Ohishi N., Nelle E., Hochstrasser R., Yabuta Y. (2012). Characterization of vitamin B₁₂compounds in the wild edible mushrooms black trumpet (*Craterellus cornucopioides*) and golden chanterelle (*Cantharellus cibarius*). J. Nutr. Sci. Vitaminol..

[105] Kosanić M., Ranković B., Stanojković T., Radović-Jakovljević M., Ćirić A., Grujičić D., Milošević-Djordjević O. (2019). *Craterellus cornucopioides* edible mushroom as source of biologically active compounds. Nat. Product Commun..

[106] Liu Y.-T., Sun J., Luo Z.-Y., Rao S.-Q., Su Y.-J., Xu R.-R., Yang Y.-J. (2012). Chemical composition of five wild edible mushrooms collected from Southwest China and their antihyperglycemic and antioxidant activity. Food Chem. Toxicol..

[107] O’Callaghan Y.C., O’Brien N.M., Kenny O., Harrington T., Brunton N., Smyth T.J. (2015). Anti-inflammatory effects of wild Irish mushroom extracts in RAW264.7 mouse macrophage cells. J. Med. Food.

[108] Vamanu E., Nita S. (2014). Biological activity of fluidized bed ethanol extracts from several edible mushrooms. Food Sci. Biotechnol..

[109] Guo H., Feng T., Li Z.-H., Liu J.-K. (2012). Four new compounds from the Basidiomycete *Craterellus odoratus*. J. Asian Nat. Prod. Res..

[110] Sanmee R., Dell B., Lumyong P., Izumori K., Lumyong S. (2003). Nutritive value of popular wild edible mushrooms from Northern Thailand. Food Chem..

[111] Zhang L., Shen Y., Wang F., Leng Y., Liu J.-K. (2010). Rare merosesquiterpenoids from Basidiomycete *Craterellus odoratus* and their inhibition of 11β-hydroxysteroid dehydrogenases. Phytochemistry.

[112] Guo H., Feng T., Li Z.-H., Liu J.-K. (2012). Five new polyketides from the Basidiomycete *Craterellus odoratus*. Nat. Prod. Bioprospect..

[113] Zhang L., Yao J.-N., Bai X., Li Z.-H., Dong Z.-J., Liu J.-K. (2017). Two new 4,6-dimethyl-3,4-dihydrochromen-2-one derivatives from *Craterellus odoratus*. J. Asian Nat. Prod. Res..

[114] Zhao Z.-Z., Zhao K., Chen H.-P., Bai X., Zhang L., Liu J.-K. (2018). Terpenoids from the mushroom-associated fungus *Montagnula donacina*. Phytochemistry.

[115] Heleno S.A., Barros L., Sousa M.J., Martins A., Ferreira I.C.F.R. (2010). Tocopherols composition of Portuguese wild mushrooms with antioxidant capacity. Food Chem..

[116] Liktor-Busa E., Kovács B., Urbán E., Hohmann J., Ványolós A. (2016). Investigation of Hungarian mushrooms for antibacterial activity and synergistic effects with standard antibiotics against resistant bacterial strains. Lett. Appl. Microbiol..

[117] Tsuge N., Mori T., Hamano T., Tanaka H., Shin-ya K., Seto H. (1999). Cinnatriacetins A and B, new antibacterial triacetylene derivatives from the fruiting bodies of *Fistulina hepatica*. J. Antibiot..

[118] Ribeiro B., Valentão P., Baptista P., Seabra R.M., Andrade P.B. (2007). Phenolic compounds, organic acids profiles and antioxidative properties of beefsteak fungus (*Fistulina hepatica*). Food Chem. Toxicol..

[119] Froufe H.J.C., Abreu R.M.V., Ferreira I.C.F.R. (2011). QCAR models to predict wild mushrooms radical scavenging activity, reducing power and lipid peroxidation inhibition. Chemom. Intell. Lab. Syst..

[120] Ribeiro B., Andrade P.B., Silva B.M., Baptista P., Seabra R.M., Valentão P. (2008). Comparative study on free amino acid composition of wild edible mushroom species. J. Agric. Food Chem..

[121] Ribeiro B., Guedes de Pinho P., Andrade P.B., Baptista P., Valentão P. (2009). Fatty acid composition of wild edible mushrooms species: A comparative study. Microchem. J..

[122] Wu S., Krings U., Zorn H., Berger R.G. (2005). Volatile compounds from the fruiting bodies of beefsteak fungus *Fistulina hepatica* (Schaeffer: Fr.) Fr. Food Chem..

[123] de Pinho P.G., Ribeiro B., Gonçalves R.F., Baptista P., Valentão P., Seabra R.M., Andrade P.B. (2008). Correlation between the pattern volatiles and the overall aroma of wild edible mushrooms. J. Agric. Food Chem..

[124] Wu S., Zorn H., Krings U., Berger R.G. (2007). Volatiles from submerged and surface-cultured beefsteak fungus, *Fistulina hepatica*. Flavour Fragr. J..

[125] Alves M.J., Ferreira I.C.F.R., Lourenço I., Castro A., Pereira L., Martins A., Pintado M. (2014). Wild mushroom extracts potentiate the action of standard antibiotics against multiresistant bacteria. J. Appl. Microbiol..

[126] Alves M.J., Fernandes Â., Barreira J.C.M., Lourenço I., Fernandes D., Moura A., Ribeiro A.R., Salgado J., Antonio A., Ferreira I.C.F.R. (2015). How gamma-rays and electron-beam irradiation would affect the antimicrobial activity of differently processed wild mushroom extracts?. J. Appl. Microbiol..

[127] Fernandes Â., Barreira J.C.M., Antonio A.L., Santos P.M.P., Martins A., Oliveira M.B.P.P., Ferreira I.C.F.R. (2013). Study of chemical changes and antioxidant activity variation induced by gamma-irradiation on wild mushrooms: Comparative study through principal component analysis. Food Res. Int..

[128] Kavishree S., Hemavathy J., Lokesh B.R., Shashirekha M.N., Rajarathnam S. (2008). Fat and fatty acids of Indian edible mushrooms. Food Chem..

[129] Takahashi A., Endo T., Nozoe S. (1992). Repandiol, a new cytotoxic diepoxide from the mushrooms *Hydnum repandum* and *H. repandum var. album*. Chem. Pharm. Bull..

[130] Wang X.N., Du J.C., Tan R.X., Liu J.K. (2005). Chemical constituents of Basidiomycete *Hydnum repandum*. Chin. Tradit. Herbal Drugs.

[131] Florianowicz T. (2000). Inhibition of growth and sporulation of *Penicillium expansum* by extracts of selected basidiomycetes. Acta Soc. Bot. Pol..

[132] Yamaç M., Bilgili F. (2006). Antimicrobial activities of fruit bodies and/or mycelial cultures of some mushroom isolates. Pharm. Biol..

[133] Murcia M.A., Martínez-Tomé M., Jiménez A.M., Vera A.M., Honrubia M., Parras P. (2002). Antioxidant activity of edible fungi (truffles and mushrooms): Losses during industrial processing. J. Food Prot..

[134] Berg A., Reiber K., Dörfelt H., Walther G., Schlegel B., Gräfe U. (2000). Laccaridiones A and B, new protease inhibitors from *Laccaria amethystea*. J. Antibiot..

[135] Liu R., Zhou Z.-Y., Jiang M.-Y., Wang F., Liu J.-K. (2010). A new isoprenyl phenyl ether riboside from the culture of Basidiomycete *Laccaria amethystea*. J. Asian Nat. Prod. Res..

[136] He L., Liang G., Guoying Z., Jun-ang L. (2011). Analysis of genetic diversity of *Lactarius hatsudake* in South China. Can. J. Microbiol..

[137] Clericuzio M., Gilardoni G., Malagòn O., Vidari G., Finzi P.V. (2008). Sesquiterpenes of *Lactarius* and *Russula* (mushrooms): An update. Nat. Prod. Commun..

[138] Miyazawa M., Kawauchi Y., Matsuda N. (2010). Character impact odorants from wild mushroom (*Lactarius hatsudake*) used in Japanese traditional food. Flavour Fragr. J..

[139] Gao J.-M., Wang M., Liu L.-P., Wei G.-H., Zhang A.-L., Draghici C., Konishi Y. (2007). Ergosterol peroxides as phospholipase A_2_ inhibitors from the fungus *Lactarius hatsudake*. Phytomedicine.

[140] Zhang A.-L., Liu L.-P., Wang M., Gao J.-M. (2007). Bioactive ergosterol derivatives isolated from the fungus *Lactarius hatsudake*. Chem. Nat. Compd..

[141] Fang L.Z., Shao H.J., Yang W.Q., Liu J.K. (2006). Two new azulene pigments from the fruiting bodies of the Basidiomycete *Lactarius hatsudake*. Helv. Chim. Acta.

[142] Xu G.-H., Kim J.W., Ryoo I.-J., Choo S.-J., Kim Y.-H., Seok S.-J., Ahn J.-S., Yoo I.-D. (2010). Lactariolines A and B: New guaiane sesquiterpenes with a modulatory effect on interferon-γ production from the fruiting bodies of *Lactarius hatsudake*. J. Antibiot..

[143] Kang H.-S., Ji S.-A., Park S.-H., Kim J.-P. (2017). Lepistatins A-C, chlorinated sesquiterpenes from the cultured Basidiomycete *Lepista sordida*. Phytochemistry.

[144] Thongbai B., Wittstein K., Richter C., Miller S.L., Hyde K.D., Thongklang N., Klomklung N., Chukeatirote E., Stadler M. (2017). Successful cultivation of a valuable wild strain of *Lepista sordida* from Thailand. Mycol. Prog..

[145] Luo Q., Sun Q., Wu L., Yang Z. (2012). Structural characterization of an immunoregulatory polysaccharide from the fruiting bodies of *Lepista sordida*. Carbohydr. Polym..

[146] Miao S., Mao X., Pei R., Miao S., Xiang C., Lv Y., Yang X., Sun J., Jia S., Liu Y. (2013). Antitumor activity of polysaccharides from *Lepista sordida* against laryngocarcinoma *in vitro* and *in vivo*. Int. J. Biol. Macromol..

[147] Miao S., Mao X., Pei R., Miao S., Xiang C., Lv Y., Yang X., Sun J., Jia S., Liu Y. (2013). *Lepista sordida* polysaccharide induces apoptosis of Hep-2 cancer cells via mitochondrial pathway. Int. J. Biol. Macromol..

[148] Zhong W., Liu N., Xie Y., Zhao Y., Song X., Zhong W. (2013). Antioxidant and anti-aging activities of mycelial polysaccharides from *Lepista sordida*. Int. J. Biol. Macromol..

[149] Mazur X., Becker U., Anke T., Sterner O. (1996). Two new bioactive diterpenes from *Lepista sordida*. Phytochemistry.

[150] Chen X.-L., Wu M., Ti H.-H., Wei X.-Y., Li T.-H. (2011). Three new 3,6-dioxygenated diketopiperazines from the Basidiomycete *Lepista sordida*. Helv. Chim. Acta.

[151] Choi J.-H., Abe N., Tanaka H., Fushimi K., Nishina Y., Morita A., Kiriiwa Y., Motohashi R., Hashizume D., Koshino H. (2010). Plant-growth regulator, imidazole-4-carboxamide, produced by the fairy ring forming fungus *Lepista sordida*. J. Agric. Food Chem..

[152] Choi J.-H., Fushimi K., Abe N., Tanaka H., Maeda S., Morita A., Hara M., Motohashi R., Matsunaga J., Eguchi Y. (2010). Disclosure of the “fairy” of fairy-ring-forming fungus *Lepista sordida*. ChemBioChem.

[153] Choi J.-H., Wu J., Sawada A., Takeda S., Takemura H., Yogosawa K., Hirai H., Kondo M., Sugimoto K., Asakawa T. (2018). *N*-glucosides of fairy chemicals, 2-azahypoxanthine and 2-aza-8-oxohypoxanthine, in rice. Org. Lett..

[154] Ma G., Zhang L., Yamawaki K., Yahata M., Choi J.-H., Kawagishi H., Kato M. (2015). Fairy chemicals, 2-azahypoxanthine and 2-aza-8-oxohypoxanthine, regulate carotenoid accumulation in citrus juice sacs *in vitro*. J. Agric. Food Chem..

[155] Ito A., Choi J.-H., Wu J., Tanaka H., Hirai H., Kawagishi H. (2017). Plant growth inhibitors from the culture broth of fairy ring-forming fungus *Lepista sordida*. Mycoscience.

[156] Nedelcheva D., Antonova D., Tsvetkova S., Marekov I., Momchilova S., Nikolova-Damyanova B., Gyosheva M. (2007). TLC and GC-MS probes into the fatty acid composition of some *Lycoperdaceae* mushrooms. J. Liq. Chromatogr. Relat. Technol..

[157] Akatin M.Y. (2013). Characterization of a β-glucosidase from an edible mushroom, *Lycoperdon pyriforme*. Int. J. Food Prop..

[158] KÖpcke B., Mayer A., Anke H., Sterner O. (1999). Bioactive azo- and azoxyformamides from *Lycoperdon pyriforme* (Schaeff. Ex Pers.). Nat. Prod. Lett..

[159] Dyakov M.Y., Kamzolkina O.V., Shtaer O.V., Bis’ko N.A., Poedinok N.L., Mikhailova O.B., Tikhonova O.V., Tolstikhina T.E., Vasil’eva B.F., Efremenkova O.V. (2011). Morphological characteristics of natural strains of certain species of Basidiomycetes and biological analysis of antimicrobial activity under submerged cultural conditions. Microbiology.

[160] Klančnik A., Megušar P., Sterniša M., Jeršek B., Bucar F., Smole Možina S., Kos J., Sabotič J. (2017). Aqueous extracts of wild mushrooms show antimicrobial and antiadhesion activities against bacteria and fungi. Phytother. Res..

[161] Prasad R., Varshney V.K., Harsh N.S.K., Kumar M. (2015). Antioxidant capacity and total phenolics content of the fruiting bodies and submerged cultured mycelia of sixteen higher basidiomycetes mushrooms from India. Int. J. Med. Mushrooms..

[162] Hosoe T., Sakai H., Ichikawa M., Itabashi T., Ishizaki T., Kawai K.-I. (2007). Lepidepyrone, a new gamma-pyrone derivative, from *Neolentinus lepideus*, inhibits hyaluronidase. J. Antibiot..

[163] Yoon K.N., Lee J.S., Kim H.Y., Lee K.R., Shin P.G., Cheong J.C., Yoo Y.B., Alam N., Ha T.M., Lee T.S. (2011). Appraisal of antihyperlipidemic activities of *Lentinus lepideus* in hypercholesterolemic rats. Mycobiology.

[164] Hanssen H.-P. (1982). Sesquiterpene hydrocarbons from *Lentinus lepideus*. Phytochemistry.

[165] Hanssen H.-P. (1985). Sesquiterpene alcohols from *Lentinus lepideus*. Phytochemistry.

[166] Li Y., Bao L., Song B., Han J., Li H., Zhao F., Liu H. (2013). A new benzoquinone and a new benzofuran from the edible mushroom *Neolentinus lepideus* and their inhibitory activity in NO production inhibition assay. Food Chem..

[167] Ishihara A., Ide Y., Bito T., Ube N., Endo N., Sotome K., Maekawa N., Ueno K., Nakagiri A. (2018). Novel tyrosinase inhibitors from liquid culture of *Neolentinus lepideus*. Biosci. Biotechnol. Biochem..

[168] Yoon K.N., Alam N., Lee K.R., Shin P.G., Cheong J.C., Yoo Y.B., Lee T.S. (2011). Antioxidant and antityrosinase activities of various extracts from the fruiting bodies of *Lentinus lepideus*. Molecules.

[169] Jung Y.-S., Yang B.-K., Jeong Y.-T., Islam R., Kim S.-M., Song C.-H. (2008). Immunomodulating activities of water-soluble exopolysaccharides obtained from submerged culture of *Lentinus lepideus*. J. Microbiol. Biotechnol..

[170] Doskocil I., Havlik J., Verlotta R., Tauchen J., Vesela L., Macakova K., Opletal L., Kokoska L., Rada V. (2016). *In vitro* immunomodulatory activity, cytotoxicity and chemistry of some central European polypores. Pharm. Biol..

[171] Yang R.-H., Bao D.-P., Guo T., Li Y., Ji G.-Y., Ji K.-P., Tan Q. (2019). Bacterial profiling and dynamic succession analysis of *Phlebopus portentosus* casing soil using MiSeq sequencing. Front. Microbiol..

[172] Kumla J., Danell E., Lumyong S. (2015). Improvement of yield for a tropical black bolete, *Phlebopus portentosus*, cultivation in Northern Thailand. Mycoscience.

[173] Kaewnarin K., Suwannarach N., Kumla J., Lumyong S. (2016). Phenolic profile of various wild edible mushroom extracts from Thailand and their antioxidant properties, anti-tyrosinase and hyperglycaemic inhibitory activities. J. Funct. Foods..

[174] Sun Z., Hu M., Sun Z., Zhu N., Yang J., Ma G., Xu X. (2018). Pyrrole alkaloids from the edible mushroom *Phlebopus portentosus* with their bioactive activities. Molecules.

[175] Chon S.-H., Yang E.-J., Lee T., Song K.-S. (2016). β-secretase (BACE1) inhibitory and neuroprotective effects of *p*-terphenyls from *Polyozellus multiplex*. Food Funct..

[176] Hwang J.S., Song K.S., Kim W.G., Lee T.H., Koshino H., Yoo I.D. (1997). Polyozellin, a new inhibitor of prolyl endopeptidase from *Polyozellus multiplex*. J. Antibiot..

[177] Takahashi S., Kawano T., Nakajima N., Suda Y., Usukhbayar N., Kimura K.-i., Koshino H. (2018). Synthesis of polyozellin, a prolyl oligopeptidase inhibitor, and its structural revision. Bioorg. Med. Chem. Lett..

[178] Kwak J.-Y., Rhee I.-K., Lee K.-B., Hwang J.-S., Yoo I.-D., Song K.-S. (1999). Thelephoric acid and kynapcin-9 in mushroom *Polyozellus multiflex* inhibit prolyl endopeptidase *in vitro*. J. Microbiol. Biotechnol..

[179] Lee H.J., Rhee I.K., Lee K.B., Yoo I.D., Song K.S. (2000). Kynapcin-12, a new *p*-terphenyl derivative from *Polyozellus multiplex*, inhibits prolyl endopeptidase. J. Antibiot..

[180] Takahashi S., Yoshida A., Uesugi S., Hongo Y., Kimura K.-i., Matsuoka K., Koshino H. (2014). Structural revision of kynapcin-12 by total synthesis, and inhibitory activities against prolyl oligopeptidase and cancer cells. Bioorg. Med. Chem. Lett..

[181] Nagasawa I., Kaneko A., Suzuki T., Nishio K., Kinoshita K., Shiro M., Koyama K. (2014). Potential anti-angiogenesis effects of *p*-terphenyl compounds from *Polyozellus multiplex*. J. Nat. Prod..

[182] Kim S.-I., Park I.-H., Song K.-S. (2002). Kynapcin-13 and-28, new benzofuran prolyl endopeptidase inhibitors from *Polyozellus multiplex*. J. Antibiot..

[183] Song K.-S., Raskin I. (2002). A prolyl endopeptidase-inhibiting benzofuran dimer from *Polyozellus multiflex*. J. Nat. Prod..

[184] Hwang J.S., Song K.-S., Kim Y.-S., Seok S.-J., Lee T.-H., Yoo I.D. (1996). Lipid peroxidation inhibitors from *Polyozellus multiplex*. Korean J. Microbiol. Biotechnol..

[185] Lee I.-S., Nishikawa A. (2003). *Polyozellus multiplex*, a Korean wild mushroom, as a potent chemopreventive agent against stomach cancer. Life Sci..

[186] Lee D., Boo K.H., Lee J.-M., Unno T., Lee W.S., Cho M., Riu K.Z., Lee D.-S. (2013). Anti-viral activity of blue chanterelle (*Polyozellus multiplex*) that inhibits α-glucosidase. Food Sci. Biotechnol..

[187] Yang E.-J., Song K.-S. (2015). Polyozellin, a key constituent of the edible mushroom *Polyozellus multiplex*, attenuates glutamate-induced mouse hippocampal neuronal HT22 cell death. Food Funct..

[188] Kim J.H., Lee J.S., Song K.-S., Kwon C.-S., Kim Y.K., Kim J.-S. (2004). Polyozellin isolated from *Polyozellus multiplex* induces phase 2 enzymes in mouse hepatoma cells and differentiation in human myeloid leukaemic cell lines. J. Agric. Food Chem..

[189] Jeong N.-H., Lee S., Choi J.K., Choi Y.-A., Kim M.-J., Lee H.-S., Shin T.-Y., Jang Y.H., Song K.-S., Kim S.-H. (2020). Polyozellin alleviates atopic dermatitis-like inflammatory and pruritic responses in activated keratinocytes and mast cells. Biomed. Pharmacother..

[190] Jin X.Y., Lee S.H., Kim J.Y., Zhao Y.-Z., Park E.-J., Lee B.-S., Nan J.-X., Song K.-S., Ko G., Sohn D.H. (2006). Polyozellin inhibits nitric oxide production by down-regulating LPS-induced activity of NF-κB and SAPK/JNK in RAW 264.7 cells. Planta Med..

[191] Jung B., Yang E.-J., Bae J.-S. (2016). Suppressive effects of polyozellin on TGFBIp-mediated septic responses in human endothelial cells and mice. Nutr. Res..

[192] Ku S.-K., Yang E.-J., Kang H., Jung B., Bae J.-S. (2016). Inhibitory effect of polyozellin on secretory group IIA phospholipase A2. Arch. Pharm. Res..

[193] Lee S.H., Song K.-S., Sohn D.H., Seo G.S. (2011). Polyozellin blocks tumor necrosis factor α-induced interleukin 8 and matrix metalloproteinase 7 production in the human intestinal epithelial cell line HT-29. Arch. Pharm. Res..

[194] Lee W., Yang E.-J., Park D.H., Bae J.-S. (2016). Suppressive effects of polyozellin on endothelial protein C receptor shedding via inhibiting TACE activity and MAP kinases. Fitoterapia.

[195] Yang E.-J., Ku S.-K., Lee W., Song K.-S., Bae J.-S. (2015). Inhibitory effects of polyozellin from *Polyozellus multiplex* on HMGB1-mediated septic responses. Inflamm. Res..

[196] Zhou R., Han Y.-J., Zhang M.-H., Zhang K.-R., Ng T.B., Liu F. (2017). Purification and characterization of a novel ubiquitin-like antitumour protein with hemagglutinating and deoxyribonuclease activities from the edible mushroom *Ramaria botrytis*. AMB Expr..

[197] Bhanja S.K., Rout D., Patra P., Sen I.K., Nandan C.K., Islam S.S. (2014). Water-insoluble glucans from the edible fungus *Ramaria botrytis*. Bioact. Carbohydr. Diet. Fibre..

[198] Bhanja S.K., Rout D., Patra P., Nandan C.K., Behera B., Maiti T.K., Islam S.S. (2013). Structural studies of an immunoenhancing glucan of an ectomycorrhizal fungus *Ramaria botrytis*. Carbohydr. Res..

[199] Li H. (2017). Extraction, purification, characterization and antioxidant activities of polysaccharides from *Ramaria botrytis* (Pers.) Ricken. Chem. Cent. J..

[200] Yaoita Y., Satoh Y., Kikuchi M. (2007). A new ceramide from *Ramaria botrytis* (Pers.) Ricken. J. Nat. Med..

[201] Vamanu E. (2018). Bioactive capacity of some Romanian wild edible mushrooms consumed mainly by local communities. Nat. Prod. Res..

[202] Li N., Ng T.B., Wong J.H., Qiao J.X., Zhang Y.N., Zhou R., Chen R.R., Liu F. (2013). Separation and purification of the antioxidant compounds, caffeic acid phenethyl ester and caffeic acid from mushrooms by molecularly imprinted polymer. Food Chem..

[203] Luo Y., Huang Y., Yuan X., Zhang L., Zhang X., Gao P. (2017). Evaluation of fatty acid composition and antioxidant activity of wild-growing mushrooms from Southwest China. Int. J. Med. Mushrooms.

[204] Barros L., Dueñas M., Ferreira I.C.F.R., Baptista P., Santos-Buelga C. (2009). Phenolic acids determination by HPLC–DAD–ESI/MS in sixteen different Portuguese wild mushrooms species. Food Chem. Toxicol..

[205] Choi J.-H., Ozawa N., Yamakawa Y., Nagai K., Hirai H., Kawagishi H. (2011). Leccinine A, an endoplasmic reticulum stress-suppressive compound from the edible mushroom. Leccinum extremiorientale. Tetrahedron..

[206] Ito A., Wu J., Ozawa N., Choi J.-H., Hirai H., Kawagishi H. (2017). Plant growth regulators from the edible mushroom *Leccinum extremiorientale*. Mycoscience.

[207] Yang N.-N., Huang S.-Z., Ma Q.-Y., Dai H.-F., Guo Z.-K., Yu Z.-F., Zhao Y.-X. (2015). A new pyrrole alkaloid from *Leccinum extremiorientale*. Chem. Nat. Compd..

[208] Sun Z.-W., Zhang L.-X., Zhang B., Niu T.-G. (2010). Structural characterisation and antioxidant properties of polysaccharides from the fruiting bodies of *Russula virescens*. Food Chem..

[209] Zhu M.-J., Du F., Zhang G.-Q., Wang H.-X., Ng T.-B. (2013). Purification a laccase exhibiting dye decolorizing ability from an edible mushroom *Russula virescens*. Int. Biodeterior. Biodegradation.

[210] Sun Z., He Y., Liang Z., Zhou W., Niu T. (2009). Sulfation of (1→3)-β-d-glucan from the fruiting bodies of *Russula virescens* and antitumor activities of the modifiers. Carbohydr. Polym..

[211] Sun Y.-X., Liu J.-C., Yang X.-D., Kennedy J.F. (2010). Purification, structural analysis and hydroxyl radical-scavenging capacity of a polysaccharide from the fruiting bodies of *Russula virescens*. Process. Biochem..

[212] Tang J., Shao H., Liu J. (2008). Chemical constituents of *Russula virescens*. Chin. Tradit. Herbal Drugs.

[213] Hasnat M.A., Pervin M., Debnath T., Lim B.O. (2014). DNA protection, total phenolics and antioxidant potential of the mushroom *Russula virescens*. J. Food Biochem..

[214] Chen X., Zhou H., Qiu G. (2010). Chemical composition and antioxidant activity of two edible mycorrhizal fungi from South China. Asian J. Chem..

[215] Sesli E. (2007). Preliminary checklist of the macromycetes of the east and middle Black Sea regions of Turkey. Mycotaxon.

[216] Meng F., Xu P., Wang X., Huang Y., Wu L., Chen Y., Teng L., Wang D. (2017). Investigation on the immunomodulatory activities of *Sarcodon imbricatus* extracts in a cyclophosphamide (CTX)-induced immunosuppressanted mouse model. Saudi Pharm. J..

[217] Alves M.J., Ferreira I.C.F.R., Lourenço I., Costa E., Martins A., Pintado M. (2014). Wild mushroom extracts as inhibitors of bacterial biofilm formation. Pathogens.

[218] Barros L., Calhelha R.C., Vaz J.A., Ferreira I.C.F.R., Baptista P., Estevinho L.M. (2007). Antimicrobial activity and bioactive compounds of Portuguese wild edible mushrooms methanolic extracts. Eur. Food Res. Technol..

[219] Barros L., Ferreira M.-J., Queirós B., Ferreira I.C.F.R., Baptista P. (2007). Total phenols, ascorbic acid, β-carotene and lycopene in Portuguese wild edible mushrooms and their antioxidant activities. Food Chem..

[220] Zhang F.-M., Wang Y.-H., Zhao P., Yu F.-Q. (2019). A new *p*-terphenyl derivative from the fruiting bodies of *Sarcodon imbricatus* (L.) P. Karst. Nat. Prod. Res..

[221] Marcotullio M.C., Mwankie G.N.O.-M., Cossignani L., Tirillini B., Pagiotti R. (2008). Phytochemical analysis and antiradical properties of *Sarcodon imbricatus* (L.: Fr) Karsten. Nat. Prod. Commun..

[222] Sulkowska-Ziaja K., Szewczyk A., Gdula-Argasinska J., Ekiert H., Jaskiewicz J., Muszynska B. (2016). Chemical compounds of extracts from *Sarcodon imbricatus* at optimized growth conditions. Acta Mycol..

[223] Sułkowska-Ziaja K., Karczewska E., Wojtas I., Budak A., Muszyńska B., Ekiert H. (2011). Isolation and biological activities of polysaccharide fractions from mycelium of *Sarcodon imbricatus* L. P. Karst. (Basidiomycota) cultured *in vitro*. Acta. Pol. Pharm..

[224] Wang X., Chu Q., Jiang X., Yu Y., Wang L., Cui Y., Lu J., Teng L., Wang D. (2018). *Sarcodon imbricatus* polysaccharides improve mouse hematopoietic function after cyclophosphamide-induced damage via G-CSF mediated JAK2/STAT3 pathway. Cell Death Dis..

[225] Wang X., Wang Z., Wu H., Jia W., Teng L., Song J., Yang X., Wang D. (2018). *Sarcodon imbricatus* polysaccharides protect against cyclophosphamide-induced immunosuppression via regulating Nrf2-mediated oxidative stress. Int. J. Biol. Macromol..

[226] Wang S., Wang K., Chen D., Zhao L. (2014). Influence of *Sarcodon imbricatus* polysaccharide on immune function in immunosuppressive mouse. J. Chem. Pharm. Res..

[227] Wang X., Qu Y., Zhang Y., Li S., Sun Y., Chen Z., Teng L., Wang D. (2018). Antifatigue potential activity of *Sarcodon imbricatus* in acute excise-treated and chronic fatigue syndrome in mice via regulation of Nrf2-mediated oxidative stress. Oxid. Med. Cell. Longev..

[228] Pegler D., Vanhaecke M. (1994). *Termitomyces* of southeast Asia. Kew Bull..

[229] Abe T., Matsumoto T. (1979). Studies on the distribution and ecological role of termites in a lowland rain forest of West Malaysia (3) distribution and abundance of termites in pasoh forest reserve. Jap. J. Ecol..

[230] Zhao H., Wang X., Liu X., Zhang J., Wan L., Jia L. (2019). Antioxidant and hypolipidemic activities of acid-depolymerised exopolysaccharides by *Termitomyces albuminosus Oxid*. Med. Cell. Longev..

[231] Hu Y., Wang T., Yang X., Zhao Y. (2014). Analysis of compositional monosaccharides in fungus polysaccharides by capillary zone electrophoresis. Carbohydr. Polym..

[232] Zhao H., Li J., Zhang J., Wang X., Liu M., Zhang C., Jia L. (2017). Hepatoprotective and *in vitro* antioxidant effects of native depolymerised-exopolysaccharides derived from *Termitomyces albuminosus*. Sci. Rep..

[233] Hong Y., Ying T. (2019). Isolation, molecular characterization and antioxidant activity of a water-soluble polysaccharide extracted from the fruiting body of *Termitornyces albuminosus* (Berk.) Heim. Int. J. Biol. Macromol..

[234] Zhao H., Li H., Feng Y., Zhang Y., Yuan F., Zhang J., Ren H., Jia L. (2019). Mycelium polysaccharides from *Termitomyces albuminosus* attenuate CCl_4_-induced chronic liver injury via inhibiting TGFβ1/Smad3 and NF-κB signal pathways. Int. J. Mol. Sci..

[235] Hong Y., Ying T. (2019). Characterization of a chitin-glucan complex from the fruiting body of *Termitomyces albuminosus* (Berk.) Heim. Int. J. Biol. Macromol..

[236] Lu Y.-Y., Ao Z.-H., Lu Z.-M., Xu H.-Y., Zhang X.-M., Dou W.-F., Xu Z.-H. (2008). Analgesic and anti-inflammatory effects of the dry matter of culture broth of *Termitomyces albuminosus* and its extracts. J. Ethnopharmacol..

[237] De Souza R.A., Kamat N.M., Nadkarni V.S. (2018). Purification and characterisation of a sulphur rich melanin from edible mushroom *Termitomyces albuminosus* Heim. Mycology.

[238] Zheng S., Wang H., Zhang G. (2011). A novel alkaline protease from wild edible mushroom *Termitomyces albuminosus*. Acta Biochim. Pol..

[239] Mau J.-L., Chang C.-N., Huang S.-J., Chen C.-C. (2004). Antioxidant properties of methanolic extracts from *Grifola frondosa*, *Morchella esculenta* and *Termitomyces albuminosus* mycelia. Food Chem..

[240] Qi J., Ojika M., Sakagami Y. (2000). Termitomycesphins A–D, novel neuritogenic cerebrosides from the edible Chinese mushroom *Termitomyces albuminosus*. Tetrahedron.

[241] Qi J., Ojika M., Sakagami Y. (2001). Neuritogenic cerebrosides from an edible Chinese mushroom. Part 2: Structures of two additional termitomycesphins and activity enhancement of an inactive cerebroside by hydroxylation. Bioorg. Med. Chem..

[242] Qu Y., Sun K., Gao L., Sakagami Y., Kawagishi H., Ojika M., Qi J. (2012). Termitomycesphins G and H, additional cerebrosides from the edible Chinese mushroom *Termitomyces albuminosus*. Biosci. Biotechnol. Biochem..

[243] Li W., Liu Q., Li S., Zheng Y. (2019). New sesquiterpenoids from the fermented broth of *Termitomyces albuminosus* and their anti-acetylcholinesterase activity. Molecules.

[244] Mondal S., Chakraborty I., Pramanik M., Rout D., Islam S.S. (2004). Structural studies of water-soluble polysaccharides of an edible mushroom, *Termitomyces eurhizus*. A reinvestigation. Carbohydr. Res..

[245] Chakraborty I., Mondal S., Rout D., Islam S.S. (2006). A water-insoluble (1→3)-β-d-glucan from the alkaline extract of an edible mushroom *Termitomyces eurhizus*. Carbohydr. Res..

[246] Chatterjee A., Khatua S., Chatterjee S., Mukherjee S., Mukherjee A., Paloi S., Acharya K., Bandyopadhyay S.K. (2013). Polysaccharide-rich fraction of *Termitomyces eurhizus* accelerate healing of indomethacin induced gastric ulcer in mice. Glycoconj. J..

[247] Mitra P., Mandal N.C., Acharya K. (2016). Polyphenolic extract of *Termitomyces heimii*: Antioxidant activity and phytochemical constituents. J. Verbrauch. Lebensm..

[248] Manna D.K., Nandi A.K., Pattanayak M., Maity P., Tripathy S., Mandal A.K., Roy S., Tripathy S.S., Gupta N., Islam S.S. (2015). A water soluble β-glucan of an edible mushroom *Termitomyces heimii*: Structural and biological investigation. Carbohydr. Polym..

[249] Abd Malek S.N., Kanagasabapathy G., Sabaratnam V., Abdullah N., Yaacob H. (2012). Lipid components of a Malaysian edible mushroom, *Termitomyces heimii* Natarajan. Int. J. Food Prop..

[250] Karun N.C., Sridhar K.R. (2013). Occurrence and distribution of *Termitomyces* (Basidiomycota, *Agaricales*) in the Western Ghats and on the West coast of India. Czech. Mycol..

[251] Aletor V.A. (1995). Compositional studies on edible tropical species of mushrooms. Food Chem..

[252] Chandra K., Ghosh K., Ojha A.K., Islam S.S. (2009). A protein containing glucan from an edible mushroom, *Termitomyces microcarpus* (var). Nat. Prod. Commun..

[253] Chandra K., Ghosh K., Roy S.K., Mondal S., Maiti D., Ojha A.K., Das D., Mondal S., Islam S.S. (2007). A water-soluble glucan isolated from an edible mushroom *Termitomyces microcarpus*. Carbohydr. Res..

[254] Bhanja S.K., Rout D. (2017). Structural analysis of two bioactive components of an edible mushroom, *Termitomyces microcarpus*. Nat. Prod. Commun..

[255] Njue A.W., Omolo J.O., Cheplogoi P.K., Langat M.K., Mulholland D.A. (2018). Cytotoxic ergostane derivatives from the edible mushroom *Termitomyces microcarpus* (Lyophyllaceae). Biochem. Syst. Ecol..

[256] Nakalembe I., Kabasa J.D. (2012). Anti-microbial activity and biochemical constituents of two edible and medicinal mushrooms of Mid-western, Uganda. Res. J. Pharmacol..

[257] Mitra P., Mandal N.C., Acharya K. (2014). Phytochemical characteristics and free radical scavenging activity of ethanolic extract of *Termitomyces microcarpus* r. Heim. Pharm. Lett..

[258] Mitra P., Mandal N., Acharya K. (2016). Mycochemicals and antioxidant activity of polyphenol-rich fraction of *Termitomyces microcarpus*. Int. Food Res. J..

[259] Woldegiorgis A.Z., Abate D., Haki G.D., Ziegler G.R. (2014). Antioxidant property of edible mushrooms collected from Ethiopia. Food Chem..

[260] Mortimer P.E., Karunarathna S.C., Li Q., Gui H., Yang X., Yang X., He J., Ye L., Guo J., Li H. (2012). Prized edible Asian mushrooms: Ecology, conservation and sustainability. Fungal Divers..

[261] Yang W.-M., Liu J.-K., Hu L., Dong Z.-J., Wu W.-L., Chen Z.-H. (2004). Antioxidant properties of natural *p*-terphenyl derivatives from the mushroom *Thelephora ganbajun*. Z. Naturforsch. C.

[262] Wang H.X., Ng T.B. (2004). Purification of a novel ribonuclease from dried fruiting bodies of the edible wild mushroom *Thelephora ganbajun*. Biochem. Biophys. Res. Commun..

[263] Gong L.L., Meng F.J., Hou Y.C., Liu Y., Xu J.J., Zhang W.N., Chen Y. (2019). Purification, characterization, and bioactivity of two new polysaccharide fractions from *Thelephora ganbajun* mushroom. J. Food Biochem..

[264] Hu L., Gao J.M., Liu J.K. (2001). Unusual poly(phenylacetyloxy)-substituted 1,1′:4′,1″-terphenyl derivatives from fruiting bodies of the Basidiomycete *Thelephora ganbajun*. Helv. Chim. Acta.

[265] Hu L., Liu J.K. (2001). Two novel phenylacetoxylated *p*-terphenyls from *Thelephora ganbajun* Zang. Z. Naturforsch. C.

[266] Xu D.-P., Zheng J., Zhou Y., Li Y., Li S., Li H.-B. (2016). Extraction of natural antioxidants from the *Thelephora ganbajun* mushroom by an ultrasound-assisted extraction technique and evaluation of antiproliferative activity of the extract against human cancer cells. Int. J. Mol. Sci..

[267] Liu J.-K., Hu L., Dong Z.-J., Hu Q. (2004). DPPH radical scavenging activity of ten natural *p*-terphenyl derivatives obtained from three edible mushrooms indigenous to China. Chem. Biodivers..

[268] Liu J.K. (2007). Secondary metabolites from higher fungi in China and their biological activity. Drug Discov. Ther..

[269] Wu F., Zhou L.-W., Yang Z.-L., Bau T., Li T.-H., Dai Y.-C. (2019). Resource diversity of Chinese macrofungi: Edible, medicinal and poisonous species. Fungal Divers..

[270] Das D., Maiti D., Chandra K., Mondal S., Ojha A.K., Roy S.K., Ghosh K., Islam S.S. (2008). NMR and MALDI-TOFMS analysis of a heteroglycan isolated from hot water extract of edible mushroom, *volvariella bombycina*. Carbohydr. Res..

[271] Xu G.-H., Kim Y.-H., Choo S.-J., Ryoo I.-J., Zheng C.-J., Seok S.-J., Kim W.-G., Yoo I.-D. (2009). Isodeoxyhelicobasidin, a novel human neutrophil elastase inhibitor from the culture broth of *Volvariella bombycina*. J. Antibiot..

[272] Xu G.-H., Choo S.-J., Kim Y.-H., Ryoo I.-J., Seok S.-J., Ahn J.-S., Yoo I.-D. (2010). Secondary metabolites of *Volvariella bombycina* and their inhibitory effects on melanogenesis. J. Microbiol. Biotechnol..

[273] Park K.M., Kwon K.M., Lee S.H. (2015). Evaluation of the antioxidant activities and tyrosinase inhibitory property from mycelium culture extracts. Evid. Based Complementary Altern. Med..

[274] Sodhi N.S., Koh L.P., Brook B.W., Ng P.K.L. (2004). Southeast Asian biodiversity: An impending disaster. Trends Ecol. Evol..

[275] Conversation T. Even as More New Species Are Found, Southeast Asia Is in the Grip of a Biodiversity Crisis. https://theconversation.com/even-as-more-new-species-are-found-southeast-asia-is-in-the-grip-of-a-biodiversity-crisis-67700.

[276] Hughes A.C. (2017). Understanding the drivers of Southeast Asian biodiversity loss. Ecosphere.

